# Recent Duplications Dominate VQ and WRKY Gene Expansions in Six *Prunus* Species

**DOI:** 10.1155/2021/4066394

**Published:** 2021-12-17

**Authors:** Yan Zhong, Ping Wang, Xiaohui Zhang, Zong-Ming Cheng

**Affiliations:** ^1^College of Horticulture, Nanjing Agricultural University, Nanjing 210095, China; ^2^School of Life Science, Nanjing University, Nanjing 210023, China

## Abstract

Genes encoding VQ motif-containing (VQ) transcriptional regulators and WRKY transcription factors can participate separately or jointly in plant growth, development, and abiotic and biotic stress responses. In this study, 222 VQ and 645 WRKY genes were identified in six *Prunus* species. Based on phylogenetic tree topologies, the VQ and WRKY genes were classified into 13 and 32 clades, respectively. Therefore, at least 13 VQ gene copies and 32 WRKY gene copies were present in the genome of the common ancestor of the six *Prunus* species. Similar small Ks value peaks for the VQ and WRKY genes suggest that the two gene families underwent recent duplications in the six studied species. The majority of the Ka/Ks ratios were less than 1, implying that most of the VQ and WRKY genes had undergone purifying selection. Pi values were significantly higher in the VQ genes than in the WRKY genes, and the VQ genes therefore exhibited greater nucleotide diversity in the six species. Forty-one of the *Prunus* VQ genes were predicted to interact with 44 of the WRKY genes, and the expression levels of some predicted VQ-WRKY interacting pairs were significantly correlated. Differential expression patterns of the VQ and WRKY genes suggested that some might be involved in regulating aphid resistance in *P. persica* and fruit development in *P. avium*.

## 1. Introduction

During their growth and development, plants are constantly threatened by an array of adverse environmental conditions. These include abiotic stresses such as drought, heat, and salt, and biotic stresses in the form of various pathogens and insects [1–4]. Plants have numerous transcriptional regulators and transcription factors (TFs) that help them cope with such ecological stresses. These regulators and TFs also have important functions in different aspects of physiological metabolism [5, 6].

VQ motif-containing (VQ) genes have been widely identified in different plants. For example, 34 VQ family members have been identified in *Arabidopsis thaliana* [7], 75 in soybean (*Glycine max*) [8], 26 in tomato (*Solanum lycopersicum*) [9], 49 in apple [10], and 59 in tobacco (*Nicotiana tabacum* L.) [11]. VQ genes have been found to play significant roles in plant growth, development, seed germination, and resistance to abiotic and biotic stresses [12–16]. The first identified VQ gene, sigma factor binding protein1 (SIB1), was found to be involved in resistance to *Botrytis cinerea* in *A. thaliana* [12, 13], and *AtVQ14* was shown to participate in the HAIKU (IKU) pathway, regulating endosperm development and seed size [14].

The WRKY gene family is one of the largest TF superfamilies in eukaryotes [17, 18], and WRKY genes are generally more numerous than VQ genes in various species. There are 56 known WRKY genes in rose (*Rosa chinensis*) [19], 94 in sorghum (*Sorghum bicolor* [L.] Moench) [20], 104 in poplar (*Populus trichocarpa*) [21], and 112 in cotton (*Gossypium raimondii*) [22]. WRKY genes are associated with seed dormancy, flowering, lignin biosynthesis, and leaf senescence in rice (*Oryza sativa*), *Arabidopsis*, Chinese flowering cabbage (*Brassica rapa* L. ssp. *pekinensis*), poplar (*P. trichocarpa*), and wheat (*Triticum aestivum* L.) [23–28]. WRKY genes also participate extensively in plant responses to abiotic and biotic stresses [29, 30]. *VvWRKY30* and *GmWRKY12* have been shown to enhance the salt tolerance of grapevine (*Vitis vinifera*) and soybean (*G. max*), respectively [31, 32], and rice and *Arabidopsis* WRKY genes have been shown to participate in the plant immune response to pathogens [13, 33].

Interaction models of VQ and WRKY proteins have revealed that their interactions play important roles in plant immunity, growth, and development [5, 34–41]. For example, the interaction between *AtVQ10* and *AtWRKY8* reduces the damage caused by *B. cinerea* infection in *Arabidopsis* [42], and the interaction between *MaVQ5* and *MaWRKY26* enhances the survival of banana (*Musa acuminata*) under cold stress [43]. The interaction of *AtVQ20* with *AtWRKY2* and *AtWRKY34* mediates male gametophytic functions and pollen development in *Arabidopsis* [44, 45].

*Prunus* species are important members of the Rosaceae family and include various ornamental and economically important fruit trees such as *P. yedoensis*, *P. persica*, and *P. dulcis* [46–51]. Although the different species show a variety of karyotypes in nature [52], *Prunus* genomes are usually very similar in size and basic synteny [53, 54]. The chromosome numbers of the genus *Prunus* are *x* = 8, and *Prunus* species evolved from a common ancestor with *x* = 9 [55, 56]. The karyotypes of *Prunu*s plants remain highly conserved, implying close relationships among these species [54, 55]. However, no genome-wide analysis has been performed to investigate the evolutionary patterns of VQ and WRKY genes and their interactions in closely related *Prunus* species. In this study, we identified VQ and WRKY genes in the genomes of *P. yedoensis*, *P. domestica*, *P. avium*, *P. dulcis*, *P. persica*, and *P. yedoensis* var. *nudiflora*. We then analyzed the phylogenetic relationships, evolutionary patterns, predicted interaction networks, and expression profiles of the VQ and WRKY genes from these species.

## 2. Materials and Methods

### 2.1. The Identification of VQ and WRKY Genes

The whole genome sequences of *P. yedoensis*, *P. domestica*, *P. avium*, *P. dulcis*, *P. persica*, and *P. yedoensis* var. *nudiflora* were downloaded from the Genome Database for Rosaceae (GDR; https://www.rosaceae.org/). All proteins in the genomes of each species were predicted using InterProScan with default parameters. Genes encoding a VQ or WRKY domain were identified as VQ or WRKY genes based on the InterProScan predictions. For genes with multiple transcripts, only the first transcript was retained for further analysis. The amino acid sequences of the VQ domain (PF05678) and the WRKY domain (PF03106) were obtained from the Pfam database (http://pfam.xfam.org/) and used as query sequences in TBLASTN searches against all the nucleotide coding sequences (CDSs) in the six genomes (*E* value ≤ 10^−4^) [57]. The BLAST hit sequences were verified by Pfam analysis, and hits that encoded VQ or WRKY domains were considered to be members of the VQ or WRKY gene family. Finally, the VQ and WRKY candidate genes were cross-verified by InterProScan and BLAST analysis. The sequences of VQ and WRKY genes from *Fragaria vesca* were obtained from previous studies [58, 59].

### 2.2. Construction of Phylogenetic Trees and Classification of Clades

The CDSs of the two gene families from the six *Prunus* species and the outgroup *F. vesca* were converted into amino acid sequences for alignment, and then, the amino acid alignments were converted back to nucleotide alignments to construct the phylogenetic trees. The VQ and WRKY phylogenetic trees were constructed in MEGA 7 using the neighbor-joining (NJ) method with pairwise deletion, and bootstrap values were obtained from 1000 replicates [60]. The trees were divided into various clades using two criteria: (i) each clade should contain one or more genes from *F. vesca* and (ii) each clade should contain genes from five or six *Prunus* species.

### 2.3. Genetic Parameters of VQ and WRKY Genes

Alignments of the CDSs from each clade in the two phylogenetic trees were obtained using ClustalW2 based on the alignment of amino acid sequences from the six *Prunus* species [61]. MEGA 7 was then used to calculate the synonymous substitution rate (Ks), nonsynonymous substitution rate (Ka), Ka*/*Ks ratio, and genetic diversity (Pi) values in each clade of the two gene families [60]. Due to Ks saturation, Ks values larger than 1 were discarded in the subsequent analysis. The duplication times (*T*) of the VQ and WRKY genes were estimated based on a mutation rate of 9.48 × 10^−9^ point mutations per site per generation and 3 years/generation in peach: *T* = Ks/[(9.48 × 10^−9^)/3] [62]. At the same time, the frequency of sequence exchanges in VQ and WRKY genes was examined using the GENECONV program with default parameters [63].

### 2.4. Prediction of Interactions between VQ and WRKY Genes

The VQ and WRKY CDSs from *A. thaliana* were downloaded from TAIR10 (http://www.Arabidopsis.org) [64]. These *Arabidopsis* genes were used as reference genes to uncover potential interactions between VQ and WRKY genes in the six *Prunus* species. The VQ and WRKY genes were numbered based on their gene ID orders (Tables S1 and S2). The CDSs of the VQ and WRKY genes from *A. thaliana* and the six studied species were then aligned, and 12 phylogenetic trees were constructed using the methods described above. *Prunus* and *A. thaliana* genes located in the same clade with bootstrap values ≥ 50 were considered to be homologs based on the phylogenetic tree topologies. Interaction relationships between the VQ and WRKY genes in the six *Prunus* species could then be predicted based on those of their VQ-WRKY homologs in *A. thaliana* [6–9, 65, 66]. The predicted interaction networks were then visualized using Cytoscape 3.7.1 (https://cytoscape.org/) [67].

### 2.5. Expression Patterns of VQ and WRKY Genes in *P. persica* and *P. avium*

RNA sequencing (RNA-seq) data for two *P. persica* peach lines (susceptible S38 and resistant R36) infested with the green peach aphid (GPA) from 3 h to 72 h were obtained from a previous study [68]. We then analyzed these data further to examine the expression of VQ and WRKY genes. Differences in VQ and WRKY gene expression were identified based on thresholds of ∣log_2_(fold change) | ≥2 and false discovery rate (FDR) adjusted *P* value < 0.05. Expression heatmaps were created for VQ and WRKY genes that were differentially expressed between the R36 and S38 lines using the pheatmap package in RStudio (https://www.rstudio.com/) based on FPKM values (Fragments per Kilobase of transcript per Million mapped reads). The FPKM values of VQ and WRKY genes in sweet cherry (*P. avium*) from 3 to 94 days after full bloom (DAFB) were also used to create heatmaps using the same methods [69]. Pearson's correlation coefficients (PCCs) between FPKM values of VQ and WRKY genes with predicted interaction relationships were calculated using SPSS version 20.0 (IBM Corp., Armonk, NY, USA).

## 3. Results

### 3.1. VQ and WRKY Genes in the Six *Prunus* Species

A total of 222 VQ genes were identified in the six *Prunus* species: 55 in *P. yedoensis*, 70 in *P. domestica*, 25 in *P. avium*, 23 in *P. dulcis*, 26 in *P. persica*, and 23 in *P. yedoensis* var. *nudiflora* (Table 1). *P. domestica* contained the largest number of VQ genes, followed by *P. yedoensis*. The other four *Prunus* species had lower and similar VQ gene numbers. Because the *P. yedoensis* var. *nudiflora* genome assembly is approximately half the size of the *P. yedoensis* assembly, it contained approximately half as many VQ genes.

The total number of WRKY genes identified in the six *Prunus* species (645) was greater than that of VQ genes (Table 1). The number of WRKY genes in each species was also greater than the number of VQ genes. Unsurprisingly, as with the VQ genes, the largest number of WRKY genes (262) was found in *P. domestica*, and this number was twice that found in *P. yedoensis* (139*).* Very similar WRKY gene numbers were found in *P. avium* (53), *P. dulcis* (56), and *P. persica* (58). However, the number of WRKY genes in *P. yedoensis* var. *nudiflora* (77) was slightly higher than that in *P. avium*, *P. dulcis*, and *P. persica*.

The average CDS lengths of the VQ and WRKY genes in the six *Prunus* species were irregular (Table 1), and the average CDS lengths were greater for WRKY genes than for VQ genes in each species.

### 3.2. Evolutionary Events Associated with VQ and WRKY Genes in the Six *Prunus* Species

**Figure 1:**
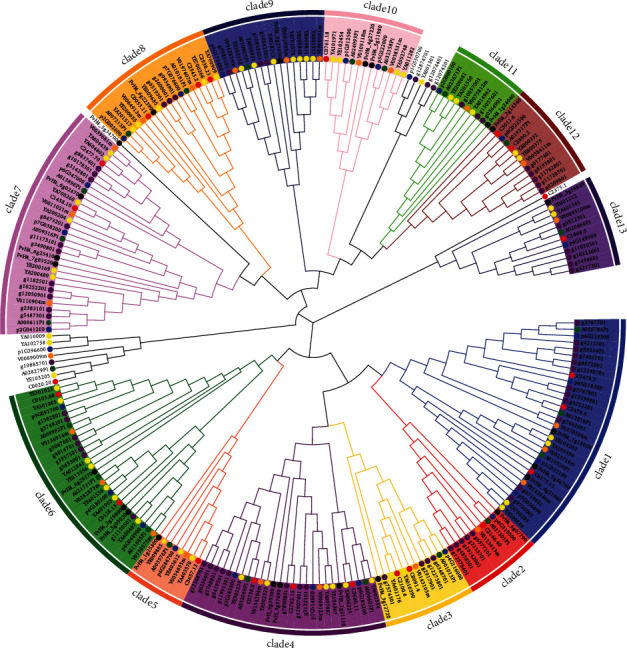
Phylogenetic tree of VQ genes from the six *Prunus* species. The colors of the filled circles denote species: yellow, *P. yedoensis*; purple, *P. domestica*; orange, *P. avium*; green, *P. dulcis*; blue, *P. persica*; and red, *P. yedoensis* var. *nudiflora.* The VQ genes of *F. vesca* are indicated by black dots. Different outer fill colors indicate different clades.

The phylogenetic tree of VQ genes could be divided into 13 clades based on the two criteria described in Materials and Methods (Figure 1), indicating that no fewer than 13 ancient VQ copies existed in the genome of the six species' common ancestor. Gene duplication and loss events were detected in each VQ clade during the evolution of the six *Prunus* species. Among all 222 VQ genes in the six species, approximately 60.81% (135/222) and 2.25% (5/222) had undergone gene duplication or gene loss, respectively (Table 2). The two species with the most VQ genes, *P. domestica* and *P. yedoensis*, also displayed the highest frequencies of gene duplication. Sixty-six VQ genes in *P. domestica* and 51 VQ genes in *P. yedoensis* were associated with 55 and 39 duplication events, respectively. These numbers of genes and duplication events were more than two times greater than those in the other four species. In general, there were very few VQ gene loss events in the six species. Two loss events occurred in *P. domestica* and *P. yedoensis* var. *nudiflora*, and one occurred in *P. yedoensis*. No loss events occurred in the remaining three species.

**Figure 2:**
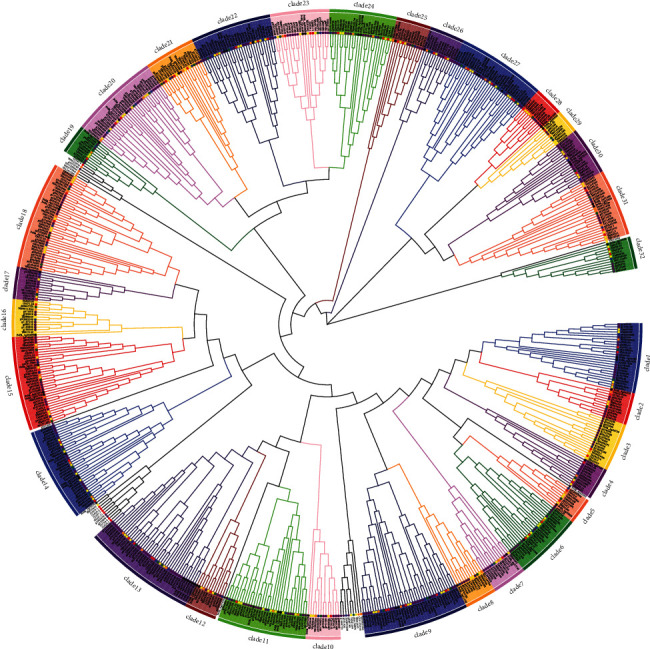
Phylogenetic tree of WRKY genes from the six *Prunus* species. The colors of the filled circles denote species: yellow, *P. yedoensis*; purple, *P. domestica*; orange, *P. avium*; green, *P. dulcis*; blue, *P. persica*; and red, *P. yedoensis* var. *nudiflora.* The WRKY genes of *F. vesca* are indicated by black dots. Different outer fill colors indicate different clades.

A phylogenetic tree was constructed using 645 WRKY genes from the six *Prunus* species and 61 WRKY genes from *F. vesca*, and the genes were classified into 32 clades (Figure 2). At least 32 ancient WRKY genes were therefore present in the ancestral species before the differentiation of the six extant *Prunus* species. Among all the WRKY genes, 66.67% (430/645) and 0.47% (3/645) were associated with gene duplication and loss events, respectively (Table 2). *P. domestica* was again associated with the most WRKY gene duplication events (223) and the largest number of WRKY genes involved in duplication (254). *P. domestica* was followed by *P. yedoensis*, with 103 duplication events and 135 duplication-related genes; these values were much lower than those of *P. domestica*. In the other four species, the number of WRKY gene duplication events ranged from 17 to 39, and the numbers of associated genes ranged from 29 to 60. Only two WRKY gene loss events were observed in *P. avium*, and one was observed in *P. yedoensis* var. *nudiflora*. No loss events were observed among the WRKY genes of the other species.

### 3.3. Duplication Times of VQ and WRKY Genes in the Six *Prunus* Species

**Figure 3:**
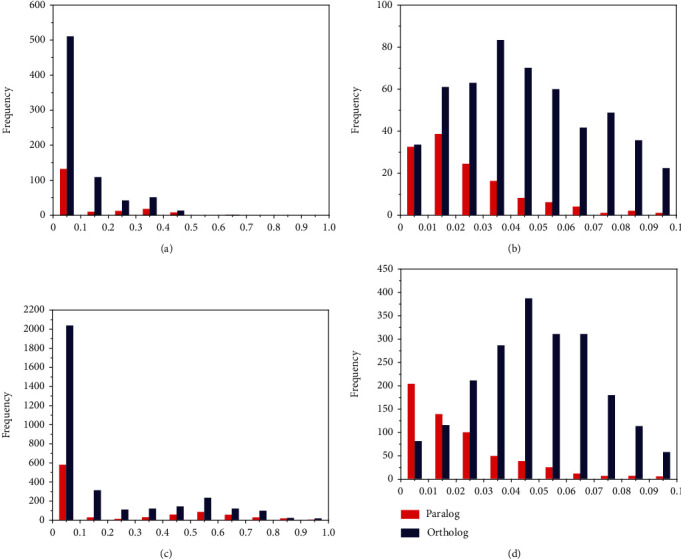
The frequency of Ks values of paralogs and orthologs of VQ genes and WRKY genes in the six *Prunus* species. The *x*-axis represents the Ks range, and the *y*-axis represents the number of gene pairs. (a) The distribution of Ks values of VQ genes within the range of 0–1.0. (b) The distribution of Ks values of VQ genes within the range of 0–0.1. (c) The distribution of Ks values of WRKY genes within the range of 0–1.0. (d) The distribution of Ks values of WRKY genes within the range of 0–0.1.

The distribution of paralog Ks values revealed the duplication times and scales of the VQ and WRKY gene families. For the VQ genes, the Ks values were not continuously distributed. Most Ks values ranged from 0 to 0.5, and almost none were found between 0.5 and 1.0 (Figure 3(a)). This result indicated that almost no VQ gene duplication events occurred at a relatively ancient stage (0.5–1.0); instead, their duplications occurred relatively recently (0–0.5). In particular, the distinct Ks peak from 0 to 0.1 clearly demonstrated that the vast majority of VQ genes in the six species arose from recent duplications. To further investigate VQ gene duplication times, Ks values in the range of 0 to 0.01 were refined into ten smaller units (Figure 3(b)). At this smaller scale, the Ks values were distributed continuously and showed peaks at 0.01–0.02 and 0–0.01. This result indicated that very recent duplications have shaped the VQ genes in the six *Prunus* species.

The Ks values of paralogous WRKY genes were distributed within the range of 0–0.9 (Figure 3(c)), indicating that duplication events have occurred continuously over the course of WRKY gene evolution. The Ks peak of the WRKY genes was also detected between 0 and 0.1, similar to that of the VQ genes. This indicated that large-scale WRKY gene duplications occurred during the same recent period as the VQ gene duplications. Within the more detailed range of 0 to 0.1, the Ks values peaked at 0–0.01 and then gradually declined from 0.01 to 0.1 (Figure 3(d)). These results showed that the evolution of the WRKY genes in the six species was also driven mainly by very recent duplications. The similar duplication times of the dominant gene expansions in the two families may be indicative of close evolutionary relationships between some VQ and WRKY genes in the six species.

### 3.4. Evolutionary Patterns of VQ and WRKY Genes in the Six *Prunus* Species

**Figure 4:**
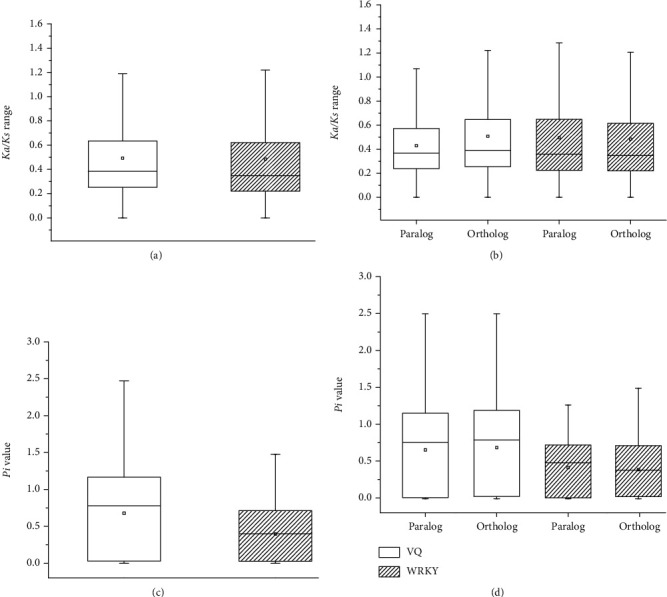
Ka*/*Ks ratios and genetic diversity (Pi) values of VQ and WRKY genes in the six *Prunus* species. (a) Ka*/*Ks ratios of VQ and WRKY genes. (b) Ka*/*Ks ratios of paralogs and orthologs among the VQ and WRKY genes. (c) Pi values of VQ and WRKY genes. (d) Pi values of paralogs and orthologs among the VQ and WRKY genes.

In general, the Ka*/*Ks ratios were greater for the VQ genes than for the WRKY genes. This suggests that fewer functional restrictions operate on the VQ genes than on the WRKY genes (Figure 4(a)). In the VQ gene family, the Ka*/*Ks ratios for both paralogs and orthologs were generally less than 1 (Figure 4(b)). Among all pairs of VQ genes in the six *Prunus* species, more than 90% (1201*/*1307) had undergone purifying selection. The remaining gene pairs had Ka*/*Ks ratio values greater than 1, indicating that a minority of the VQ genes were under positive selection. There was also a difference between the Ka*/*Ks ratios of paralogs and orthologs: orthologs had significantly higher Ka*/*Ks ratios (*t*-test, *P* < 0.01). This suggested that paralogs were subjected to more functional restrictions than orthologs among the six species. The Ka*/*Ks ratios of the WRKY gene pairs were similar to those of the VQ gene pairs; most were less than 1, but approximately 10% (673*/*6407) were greater than 1. However, the median, mean, and quartiles of the Ka*/*Ks ratios were slightly higher for the paralogs than for the orthologs (Figure 4(b)).

The Pi values were significantly greater for the VQ genes than for the WRKY genes (*t*-test, *P* < 0.01), and the VQ genes therefore displayed greater nucleotide diversity (Figure 4(c)). Among the WRKY genes, the Pi values were significantly higher for paralogs than for orthologs (*t*-test, *P* < 0.01; Figure 4(d)).

The number of sequence exchange events was more than four times greater between the WRKY genes (1039) than between the VQ genes (220), a result that reflects the larger numbers of WRKY genes in the six species (Table S3). Significantly more frequent sequence exchanges were detected between orthologs than between paralogs for both VQ (*t*-test, *P* < 0.05) and WRKY genes (*t*-test, *P* < 0.01).

### 3.5. Interactions between the VQ and WRKY Genes

Based on the topologies of the phylogenetic trees (Figures S1 and S2), *AtVQ* and *AtWRKY* homologs were identified in the six *Prunus* species (Figure S3). By analogy to the interactions of their *Arabidopsis* homologs, 41 *Prunus* VQ genes were predicted to interact with 44 WRKY genes: 8 *PyVQ* genes with 4 *PyWRKY* genes in *P. yedoensis*, 10 *PgVQs* with 9 *PgWRKYs* in *P. domestica*, 3 *PvVQs* with 6 *PvWRKYs* in *P. avium*, 4 *PaVQs* with 9 *PaWRKYs* in *P. dulcis*, 10 *PpVQs* with 7 *PpWRKYs* in *P. persica*, and 6 *PcVQs* with 9 *PcWRKYs* in *P. yedoensis* var. *nudiflora*. One-to-one, one-to-many, and many-to-many interaction relationships were all predicted between the VQ and WRKY genes of the studied *Prunus* species. The single predicted one-to-one interaction relationship was *PvVQ5*-*PvWRKY38* in *P. avium*. *PpVQ1*-*PpWRKY3*/*PpWRKY31*/*PpWRKY37* in *P. persica* and *PcVQ10*/*PcVQ17*/*PcVQ19*-*PcWRKY14* in *P. yedoensis* var. *nudiflora* were good examples of one-to-many interaction relationships. All the predicted VQ and WRKY genes in *P. yedoensis* and *P. domestica* exhibited many-to-many interaction relationships.

### 3.6. Expression Profiles of *PpVQ* and *PpWRKY* Genes in Peach during Aphid Infestation

**Figure 5:**
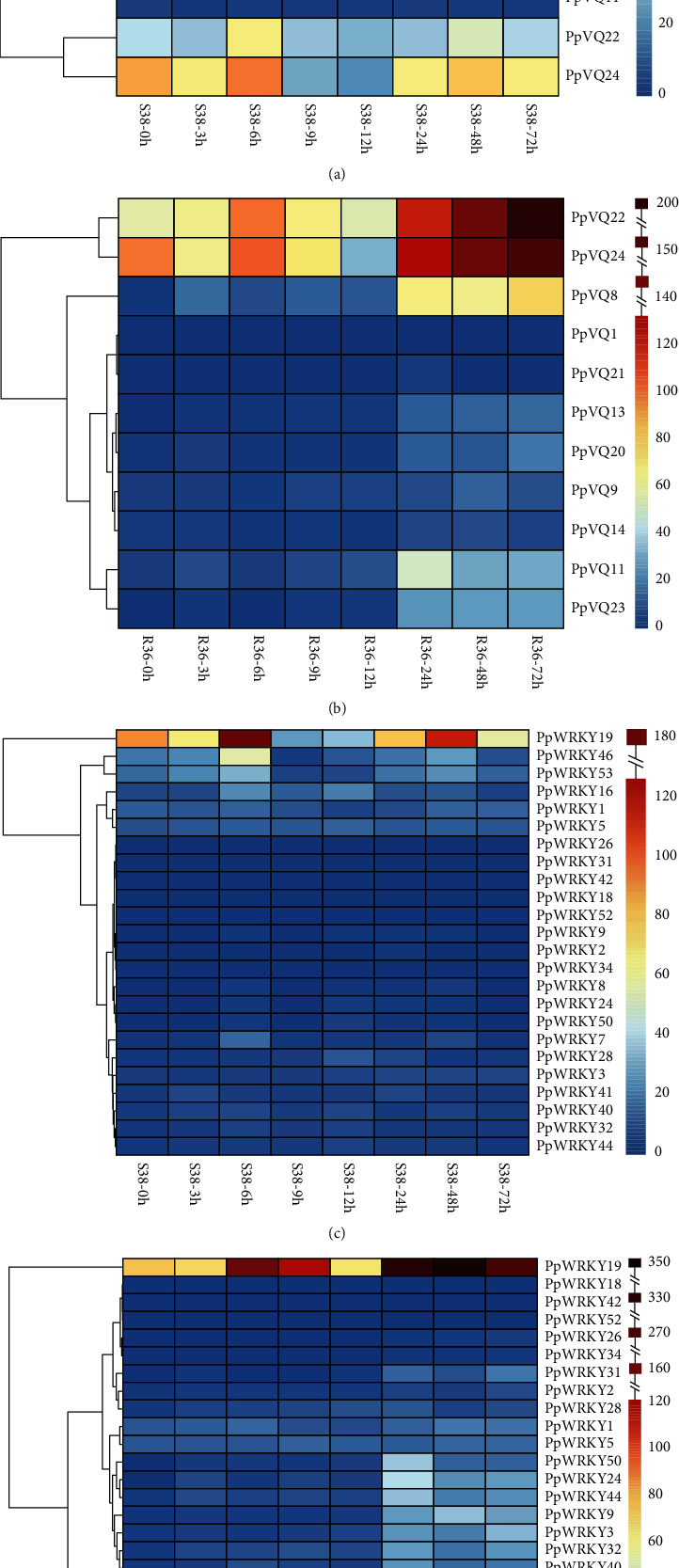
Expression of *Prunus persica* VQ and WRKY genes in two peach lines infested with green peach aphids (GPAs). (a) VQ expression in the susceptible peach line, S38. (b) VQ expression in the resistant peach line, R36. (c) WRKY expression in the susceptible peach line, S38. (d) WRKY expression in the resistant peach line, R36.

Eleven *PpVQ* and 24 *PpWRKY* genes were significantly differentially expressed between the susceptible peach line, S38, and the resistant line, R36, after GPA infestation for 0, 3, 6, 9, 12, 24, 48, and 72 h (Figure 5).

The expression levels of the differentially expressed VQ genes were significantly lower in S38 than in R36 (*t*-test, *P* < 0.01), demonstrating that these VQ genes responded more strongly to GPA infestation in the resistant line than in the susceptible line. In particular, two genes (*PpVQ22* and *PpVQ24*) had higher expression levels than the other genes in the two peach lines. They had high expression levels at 0 h, then displayed fluctuating upregulation at subsequent time points in S38 and R36. These patterns indicated that *PpVQ22* and *PpVQ24* expression continuously fluctuated in both lines during GPA infestation. In the R36 line, *PpVQ8* and *PpVQ9* also had relatively high expression levels that displayed wave-like oscillations.

The expression levels of differentially expressed WRKY genes were likewise higher in R36 than in S38. *PpWRKY19* had the highest expression level of the differentially expressed WRKY genes, and its expression also exhibited waves of upregulation from 0 to 72 h in both lines. Similar tendencies were observed for *PpWRKY46* and *PpWRKY53* in both lines and for *PpWRKY7*, *PpWRKY8*, *PpWRKY9*, *PpWRKY16*, *PpWRKY41*, *PpWRKY44*, and *PpWRKY50* in R36 only.

Although some of the same genes exhibited similar expression patterns in both lines, more VQ and WRKY genes had higher expression levels in the resistant line and may have contributed to its ability to defend against GPA infestation.

### 3.7. Expression Patterns of *PvVQ* and *PvWRKY* Genes during Sweet Cherry Exocarp Development

**Figure 6:**
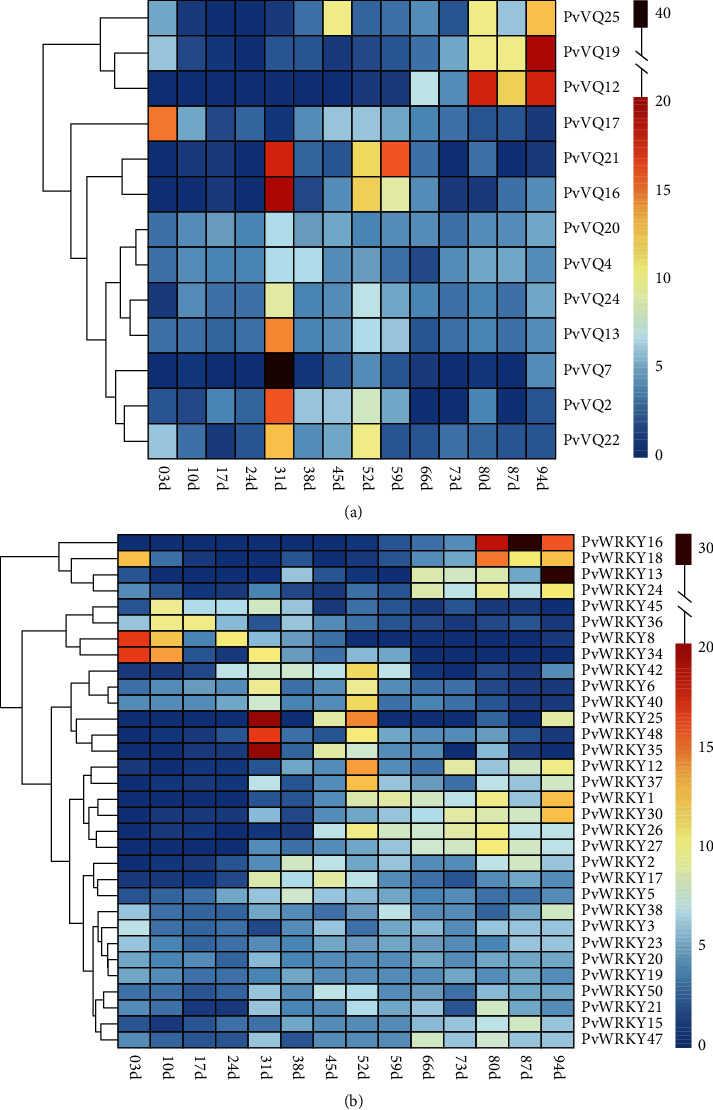
Expression profiles of *Prunus avium* (a) VQ and (b) WRKY genes in different stages of sweet cherry exocarp development. The expression is shown over time, with “d” representing days after full bloom.

Thirteen *PvVQ* genes and 32 *PvWRKY* genes were differentially expressed during the development of sweet cherry fruit exocarps from 3 to 94 DAFB. The experimental period encompassed three developmental stages: (i) 3–30 DAFB (cell division and expansion), (ii) 31–45 DAFB (seed development), and (iii) 52–94 DAFB (rapid cell expansion; Figure 6). The differentially expressed VQ genes exhibited four expression patterns from full bloom to fruit ripening. The first pattern, which was displayed by genes such as *PvVQ17*, was characterized by higher expression at 3 DAFB. The second pattern, shown by *PvVQ4* and *PvVQ20*, was characterized by similar expression at all three stages. The third pattern involved relatively high expression at 31 and 52 DAFB but lower expression at other time points. *PvVQ7*, *PvVQ16*, and *PvVQ21* displayed this pattern and may therefore be involved in the early phases of seed development and rapid cell expansion. The fourth pattern was shown by *PvVQ12*, *PvVQ19*, and *PvVQ25* and was characterized by relatively low expression at stages I and II but higher expression in the later period of stage III.

The differentially expressed WRKY genes also displayed four expression patterns. *PvWRKY8*, *PvWRKY18*, and *PvWRKY34* displayed the first pattern, with higher expression in the early phase of stage I. *PvWRKY3*, *PvWRKY19*, and *PvWRKY20* represented the second pattern, showing little variation in expression among the three stages. WRKY genes with distinctly higher expression in the early phase of stage II (e.g., *PvWRKY25* and *PvWRKY35*) or in the later phase of stage III (e.g., *PvWRKY13* and *PvWRKY16*) were assigned to the third or fourth pattern, respectively. These results indicated that some *PvVQ* and *PvWRKY* genes were differentially expressed during fruit development and maturation in sweet cherry.

### 3.8. Correlations between Related VQ and WRKY Genes

Using FPKM values of the VQ and WRKY genes in the two peach lines, PCC values were calculated between predicted VQ-WRKY interaction partners (Table 3). Ten VQ-WRKY gene pairs had significant correlations (*P* < 0.05) or very significant correlations (*P* < 0.01) in expression in the resistant peach line, R36. Nine of the significantly correlated gene pairs had significant positive correlations, but the remaining pair (*PpVQ20*-*PpWRKY37*) had a highly significant negative correlation. Only one sweet cherry gene pair (*PvVQ22-PvWRKY48*) showed a significant correlation in expression. These significantly correlated gene pairs may participate in responses to pathogen infection or in other development-related physiological activities.

## 4. Discussion

### 4.1. Gene Duplications Have Shaped the VQ and WRKY Families in Six *Prunus* Species

Gene duplication is one of the mechanisms by which genetic materials are produced for plant evolution [70]. Numerous gene families have been shaped by gene duplication in plant genomes. Examples include the VQ gene family in soybean (*G. max*) and pear (*Pyrus bretschneideri*) [8, 71] and the WRKY gene family in sorghum (*S. bicolor)* and potato (*Solanum tuberosum*) [20, 72].

In this study, at least 60% of the VQ and WRKY family members in six *Prunus* genomes were generated by gene duplication events. Gene duplication was therefore one of the most important genetic events promoting gene expansions during the evolution of these two gene families in the *Prunus* species. Although the VQ and WRKY genes of the six species originated from common ancestral VQ and WRKY copies, the different scales of the duplication events they experienced led to different numbers of VQ and WRKY genes in the six genomes. More gene duplication events were detected for the VQ and WRKY genes of *P. yedoensis* and *P. domestica* than for those of the other four species; this was the principal cause of the greater gene numbers in *P. yedoensis* and *P. domestica* (Table 2).

### 4.2. Relatively Recent Duplications Were the Main Drivers of VQ and WRKY Gene Expansions in the Six *Prunus* Species

Recent duplications have driven the expansion and evolution of plant gene families [73], including the NBS-LRR gene families in grapevine (*V. vinifera*) and poplar (*P. trichocarpa*) [74]. Most soybean VQ genes have undergone recent segmental duplications [8]. In the present study, the majority of VQ genes in the six *Prunus* species were generated by recent duplication events, as evidenced by paralogous Ks values that peaked at 0.01–0.02 and 0–0.01 (Figure 3). The most recent duplication of the VQ genes appears to have occurred approximately <6.33 million years ago (MYA), based on the mutation rate in peach (Materials and Methods). This duplication therefore arose after the divergence of the *Prunus* species, which occurred between 36 and 44 MYA [47]. Furthermore, the Ks values of WRKY gene paralogs peaked at 0–0.01, indicating that the WRKY genes of the six species began to experience recent expansion events approximately <3.16 MYA. Thus, large-scale, recent gene duplications were the dominant force driving VQ and WRKY gene expansions among the six *Prunus* species. No whole-genome duplication (WGD) events have occurred recently in *Prunus* species such as *P. persica* [49, 75–77]. Therefore, expansions of the VQ and WRKY genes may be only loosely connected with WGDs in these *Prunus* species.

### 4.3. Different Evolutionary Patterns between VQ and WRKY Gene Families in the Six *Prunus* Species

The vast majority of VQ and WRKY genes have experienced purifying selection (Figures 4(a) and 4(b); Ka/Ks < 1) to eliminate deleterious mutations and maintain gene functions during evolution [78]. These results are consistent with those for VQ genes from the genomes of three legumes and tobacco (*N. tabacum* L.) [8, 11, 79] and for the WRKY genes of *Arabidopsis*, cotton (*G. raimondii*), and rose (*R. chinensis*) [19, 22, 80]. Therefore, most members of the two gene families in the six species studied here had relatively low variation rates and conserved functions. A fraction of the VQ and WRKY genes were under positive selection (Ka/Ks > 1), which may have been related to their ability to help the plant cope with various environmental conditions [77]. Trends in Ka/Ks distribution were similar between the VQ and WRKY genes. However, Ka/Ks ratios, which are indicative of selection pressure, were higher in the VQ genes than in the WRKY genes. The VQ genes also had more significant nucleotide diversity than the WRKY genes (*t*-test, *P* < 0.01). VQ proteins interact with a variety of partners, and VQ genes therefore encode extremely diverse proteins with different primary structures [37]. Therefore, WRKY genes may show greater conservation and are more likely to be trapped by functional restrictions than VQ genes in the six *Prunus* genomes.

### 4.4. *PpVQ* and *PpWRKY* Genes Respond Together to Peach Aphid Infestation

VQ and WRKY genes have previously been reported to participate in plant disease response and defense [33, 36, 39, 42]. In the present study, 11 *PpVQ* and 24 *PpWRKY* genes were differentially expressed under GPA infestation in two peach lines (Figure 5). For example, *PpVQ24* expression responded strongly to GPA infestation; *PpVQ24* is a homolog of *AtVQ16* and *AtVQ23*, which are associated with defense against *B. cinerea* in *Arabidopsis* [13]. Likewise, *PpWRKY19*, which displayed the highest expression levels after GPA infestation, is homologous to *AtWRKY54* and *AtWRKY70*. These two *Arabidopsis* WRKY TFs negatively mediate responses to drought and necrotrophic pathogens [39, 81, 82]. These results demonstrate that *PpVQ24* and *PpWRKY19* may be involved in the GPA response in peach.

Overall, *PpVQ* and *PpWRKY* genes responded more strongly to GPA infestation in the resistant line, R36, than in the susceptible line, S38. This was consistent with the longer duration of defense in R36 than in S38 [68]. Stronger responses of VQ and WRKY genes to pathogen infection in resistant plants have been reported previously in other species. Examples include the *GmVQ* genes in soybean (*G. max*) infested with the common cutworm (*Spodoptera litura* Fabricius), *OsVQ* genes in rice (*O. sativa*) after infection with three pathogens, and WRKY genes in chrysanthemum (*Chrysanthemum morifolium*) after aphid infestation [15, 16, 35].

It is well-known that VQ proteins interact with WRKY TFs to participate jointly in plant defense against pathogens and insects. In peaches subjected to GPA infestation, the FPKM values of ten predicted interacting gene pairs (e.g., *PpVQ8*-*PpWRKY37*, *PpVQ8*-*PpWRKY44*, and *PpVQ20*-*PpWRKY37*) were significantly correlated. Interestingly, the *Arabidopsis* genes *AtVQ16* (SIB2) and *AtVQ23* (SIB1), which are homologs of *PpVQ8*, increase *B. cinerea* resistance by interacting with *AtWRKY33*, which is a *PpWRKY44* homolog [13]. We speculate that *PpVQ* and *PpWRKY* genes with highly correlated expression patterns may interact to respond together to GPA infestation in peach, and this possibility awaits further research.

## Figures and Tables

**Table 1:** Gene numbers and average lengths of nucleotide coding sequences of VQ and WRKY genes identified in the six *Prunus* species.

Species	VQ genes		WRKY genes	
	Gene number	Average length (bp)	Gene number	Average length (bp)
*P. yedoensis*	55	660.44	139	1217.68
*P. domestica*	70	581.96	262	1114.85
*P. avium*	25	730.92	53	1104.57
*P. dulcis*	23	663.26	56	1209.59
*P. persica*	26	731.42	58	1187.90
*P. yedoensis* var. *nudiflora*	23	617.22	77	1139.57
Total	222	3985.22	645	6974.16

**Table 2:** Gene duplication and gene loss of VQ and WRKY genes in the six *Prunus* species.

Gene family	Species	Gene numbers involved in duplication	Gene duplication events	Gene loss events
VQ	*P. yedoensis*	51	39	1
	*P. domestica*	66	55	2
	*P. avium*	18	11	0
	*P. dulcis*	15	9	0
	*P. persica*	17	11	0
	*P. yedoensis* var. *nudiflora*	17	10	2
	Total	184	135	5
WRKY	*P. yedoensis*	135	103	0
	*P. domestica*	254	223	0
	*P. avium*	29	17	2
	*P. dulcis*	39	23	0
	*P. persica*	41	25	0
	*P. yedoensis* var. *nudiflora*	60	39	1
	Total	558	430	3

**Table 3:** Pearson correlation coefficients of the expression levels of predicted VQ–WRKY interaction partners from *Prunus persica* and *Prunus avium.*

Species	VQ gene ID	WRKY gene ID	PCC value
*P. persica_*R36	*PpVQ1*	*PpWRKY3*	0.631
	*PpVQ1*	*PpWRKY31*	0.616
	*PpVQ1*	*PpWRKY37*	0.772^∗^
	*PpVQ4*	*PpWRKY37*	−0.222
	*PpVQ8*	*PpWRKY37*	0.950^∗∗^
	*PpVQ8*	*PpWRKY44*	0.912^∗∗^
	*PpVQ14*	*PpWRKY37*	0.817^∗^
	*PpVQ14*	*PpWRKY44*	0.810^∗^
	*PpVQ17*	*PpWRKY50*	−0.336
	*PpVQ18*	*PpWRKY15*	−0.294
	*PpVQ18*	*PpWRKY17*	0.017
	*PpVQ18*	*PpWRKY37*	−0.200
	*PpVQ20*	*PpWRKY15*	0.290
	*PpVQ20*	*PpWRKY17*	−0.333
	*PpVQ20*	*PpWRKY37*	−0.850^∗∗^
	*PpVQ21*	*PpWRKY15*	−0.171
	*PpVQ21*	*PpWRKY17*	−0.183
	*PpVQ21*	*PpWRKY37*	0.616
	*PpVQ22*	*PpWRKY3*	0.904^∗∗^
	*PpVQ22*	*PpWRKY31*	0.904^∗∗^
	*PpVQ22*	*PpWRKY37*	0.794^∗^
	*PpVQ22*	*PpWRKY44*	0.688
	*PpVQ24*	*PpWRKY37*	0.750^∗^
	*PpVQ24*	*PpWRKY44*	0.671
*P. persica_*S38	*PpVQ1*	*PpWRKY3*	0.090
	*PpVQ1*	*PpWRKY31*	0.702
	*PpVQ1*	*PpWRKY37*	0.364
	*PpVQ4*	*PpWRKY37*	−0.332
	*PpVQ8*	*PpWRKY37*	−0.214
	*PpVQ8*	*PpWRKY44*	0.683
	*PpVQ14*	*PpWRKY37*	−0.105
	*PpVQ14*	*PpWRKY44*	−0.432
	*PpVQ17*	*PpWRKY50*	0.613
	*PpVQ18*	*PpWRKY15*	−0.362
	*PpVQ18*	*PpWRKY17*	NA
	*PpVQ18*	*PpWRKY37*	−0.494
	*PpVQ20*	*PpWRKY15*	−0.020
	*PpVQ20*	*PpWRKY17*	NA
	*PpVQ20*	*PpWRKY37*	0.061
	*PpVQ21*	*PpWRKY15*	−0.062
	*PpVQ21*	*PpWRKY17*	NA
	*PpVQ21*	*PpWRKY37*	0.384
	*PpVQ22*	*PpWRKY3*	−0.239
	*PpVQ22*	*PpWRKY31*	−0.699
	*PpVQ22*	*PpWRKY37*	−0.338
	*PpVQ22*	*PpWRKY44*	0.190
	*PpVQ24*	*PpWRKY37*	−0.335
	*PpVQ24*	*PpWRKY44*	−0.202
*P. avium*	*PvVQ17*	*PvWRKY48*	−0.282
	*PvVQ22*	*PvWRKY48*	0.827^∗∗^

^∗^Significant correlation at the 0.05 level. ^∗∗^Significant correlation at the 0.01 level.

## Data Availability

The data used to support the findings of this study are available from the corresponding author upon request.

## Supplementary Materials

Supplementary 1 Table S1: the correspondence between gene IDs and VQ names in the six *Prunus* species.

Supplementary 2 Table S2: the correspondence between gene IDs and WRKY names in the six *Prunus* species.

Supplementary 3 Table S3: the sequence exchange events of VQ and WRKY genes in the six *Prunus* species.

Supplementary 4 Figure S1: phylogenetic trees of VQ genes from *Arabidopsis* and the six *Prunus* species. (a) *P. yedoensis*. (b) *P. domestica*. (c) *P. avium*. (d) *P. dulcis*. (e) *P. persica*. (f) *P. yedoensis* var. *nudiflora*. The square line indicates homologous VQ genes of *A. thaliana* and *Prunus* species in the same clade with bootstrap values ≥ 50.

Supplementary 5 Figure S2: phylogenetic trees of WRKY genes from *Arabidopsis* and the six *Prunus* species. (a) *P. yedoensis*. (b) *P. domestica*. (c) *P. avium*. (d) *P. dulcis*. (e) *P. persica*. (f) *P. yedoensis* var. *nudiflora*. The square line indicates homologous WRKY genes of *A. thaliana* and *Prunus* species in the same clade with bootstrap values ≥ 50.

Supplementary 6 Figure S3: predicted interaction networks of VQ and WRKY proteins from the six *Prunus* species. (a) *P. yedoensis*. (b) *P. domestica*. (c) *P. avium*. (d) *P. dulcis*. (e) *P. persica*. (f) *P. yedoensis* var. *nudiflora*.

## References

[1] Banerjee A., Roychoudhury A. (2015). WRKY proteins: signaling and regulation of expression during abiotic stress responses. *ScientificWorldJournal*.

[2] Bai Y. L., Kissoudis C., Yan Z., Visser R. G. F., van der Linden G. (2018). Plant behaviour under combined stress: tomato responses to combined salinity and pathogen stress. *Plant Journal*.

[3] Jiang J. J., Ma S. H., Ye N. H., Jiang M., Cao J., Zhang J. (2017). WRKY transcription factors in plant responses to stresses. *Journal of Integrative Plant Biology*.

[4] Vurukonda S. S. K. P., Vardharajula S., Shrivastava M., SkZ A. (2016). Enhancement of drought stress tolerance in crops by plant growth promoting rhizobacteria. *Microbiological Research*.

[5] Chi Y., Yang Y., Zhou Y. (2013). Protein-protein interactions in the regulation of WRKY transcription factors. *Molecular Plant*.

[6] Wang M., Vannozzi A., Wang G. (2015). A comprehensive survey of the grapevine VQ gene family and its transcriptional correlation with WRKY proteins. *Frontiers in Plant Science*.

[7] Cheng Y., Zhou Y., Yang Y. (2012). Structural and functional analysis of VQ motif-containing proteins in Arabidopsis as interacting proteins of WRKY transcription factors. *Plant Physiology*.

[8] Wang Y. B., Jiang Z. F., Li Z. X. (2019). Genome-wide identification and expression analysis of theVQgene family in soybean (glycine max). *PeerJ*.

[9] Ding H. D., Yuan G. B., Mo S. R. (2019). Genome-wide analysis of the plant-specific VQ motif-containing proteins in tomato (Solanum lycopersicum_ ) and characterization of SlVQ6 in thermotolerance. *Plant Physiology and Biochemistry*.

[10] Dong Q. L., Zhao S., Duan D. Y. (2018). Structural and functional analyses of genes encoding VQ proteins in apple. *Plant Science*.

[11] Liu C. H., Liu H., Zhou C. Y., Timko M. P. (2020). Genome-wide identification of the VQ protein gene family of tobacco (Nicotiana tabacum L.) and analysis of its expression in response to phytohormones and abiotic and biotic stresses. *Genes (Basel)*.

[12] Morikawa K., Shiina T., Murakami S., Toyoshima Y. (2002). Novel nuclear-encoded proteins interacting with a plastid sigma factor, Sig1, in Arabidopsis thaliana. *FEBS Letters*.

[13] Lai Z. B., Li Y., Wang F. (2011). Arabidopsis sigma factor binding proteins are activators of the WRKY33 transcription factor in plant defense. *Plant Cell*.

[14] Wang A. H., Garcia D., Zhang H. Y. (2010). The VQ motif protein IKU1 regulates endosperm growth and seed size in Arabidopsis. *Plant Journal*.

[15] Li X., Qin R., Du Q. (2020). Knockdown of GmVQ58 encoding a VQ motif-containing protein enhances soybean resistance to the common cutworm (Spodoptera litura Fabricius). *Journal of Experimental Botany*.

[16] Li N., Li X., Xiao J., Wang S. (2014). Comprehensive analysis of VQ motif-containing gene expression in rice defense responses to three pathogens. *Plant Cell Reports*.

[17] Jin J. P., Zhang H., Kong L., Gao G., Luo J. (2014). PlantTFDB 3.0: a portal for the functional and evolutionary study of plant transcription factors. *Nucleic Acids Research*.

[18] Zhang Y. J., Wang L. J. (2005). The WRKY transcription factor superfamily: its origin in eukaryotes and expansion in plants. *BMC Evolutionary Biology*.

[19] Liu X. T., Li D. D., Zhang S. Y., Xu Y., Zhang Z. (2019). Genome-wide characterization of the rose (Rosa chinensis) WRKY family and role of RcWRKY41 in gray mold resistance. *BMC Plant Biology*.

[20] Baillo E. H., Hanif M. S., Guo Y. H., Zhang Z., Xu P., Algam S. A. (2020). Genome-wide identification of WRKY transcription factor family members in sorghum (Sorghum bicolor(L.) moench). *PLoS One*.

[21] He H. S., Dong Q., Shao Y. H. (2012). Genome-wide survey and characterization of the WRKY gene family in Populus trichocarpa. *Plant Cell Reports*.

[22] Ding M. Q., Chen J. D., Jiang Y. R. (2015). Genome-wide investigation and transcriptome analysis of the WRKY gene family in Gossypium. *Molecular Genetics and Genomics*.

[23] Zhou C., Lin Q., Lan J. (2020). WRKY transcription factor OsWRKY29 represses seed dormancy in rice by weakening abscisic acid response. *Frontiers in Plant Science*.

[24] Li W., Wang H. P., Yu D. Q. (2016). _Arabidopsis_ WRKY Transcription Factors WRKY12 and WRKY13 Oppositely Regulate Flowering under Short-Day Conditions. *Molecular Plant*.

[25] Yang L., Zhao X., Yang F., Fan D., Jiang Y., Luo K. (2016). PtrWRKY19, a novel WRKY transcription factor, contributes to the regulation of pith secondary wall formation in _Populus trichocarpa. *Scientific Reports*.

[26] Chen L. G., Xiang S. Y., Chen Y. L., Li D., Yu D. (2017). Arabidopsis WRKY45 Interacts with the DELLA Protein RGL1 to Positively Regulate Age-Triggered Leaf Senescence. *Molecular Plant*.

[27] Fan Z. Q., Tan X. L., Shan W., Kuang J. F., Lu W. J., Chen J. Y. (2017). BrWRKY65, a WRKY transcription factor, is involved in regulating three leaf senescence-associated genes in Chinese flowering cabbage. *International Journal of Molecular Sciences*.

[28] Zhao L., Zhang W., Song Q. (2020). A WRKY transcription factor, TaWRKY40-D, promotes leaf senescence associated with jasmonic acid and abscisic acid pathways in wheat. *Plant Biology*.

[29] Li R., Zhang J., Li J. C. (2015). Prioritizing plant defence over growth through WRKY regulation facilitates infestation by non-target herbivores. *eLife*.

[30] Birkenbihl R. P., Kracher B., Ross A., Kramer K., Finkemeier I., Somssich I. E. (2018). Principles and characteristics of the Arabidopsis WRKY regulatory network during early MAMP-triggered immunity. *Plant Journal*.

[31] Zhu D., Hou L. X., Xiao P. L., Guo Y., Deyholos M. K., Liu X. (2019). VvWRKY30_, a grape WRKY transcription factor, plays a positive regulatory role under salinity stress. *Plant Science*.

[32] Shi W. Y., du Y. T., Ma J. (2018). The WRKY transcription factor GmWRKY12 confers drought and salt tolerance in soybean. *International Journal of Molecular Sciences*.

[33] Lee H., Cha J., Choi C. (2018). Rice WRKY11 plays a role in pathogen defense and drought tolerance. *Rice*.

[34] Yao D. M., Zou C., Shu Y. N., Liu S. S. (2021). WRKY transcription factors in Nicotiana tabacum modulate plant immunity against whitefly via interacting with MAPK cascade pathways. *Insects*.

[35] Li P., Song A., Gao C. (2015). The over-expression of a chrysanthemum _WRKY_ transcription factor enhances aphid resistance. *Plant Physiology and Biochemistry*.

[36] Wang H., Hu Y., Pan J., Yu D. (2015). Arabidopsis VQ motif-containing proteins VQ12 and VQ29 negatively modulate basal defense against Botrytis cinerea. *Scientific Reports*.

[37] Jing Y., Lin R. (2015). The VQ motif-containing protein family of plant-specific transcriptional regulators. *Plant Physiology*.

[38] Hu P., Zhou W., Cheng Z. W., Fan M., Wang L., Xie D. (2013). JAV1 Controls Jasmonate-Regulated Plant Defense. *Molecular Cell*.

[39] Li J., Zhong R., Palva E. T. (2017). WRKY70 and its homolog WRKY54 negatively modulate the cell wall-associated defenses to necrotrophic pathogens in Arabidopsis. *PLoS One*.

[40] Besseau S., Li J., Palva E. T. (2012). WRKY54 and WRKY70 co-operate as negative regulators of leaf senescence in Arabidopsis thaliana. *Journal of Experimental Botany*.

[41] Lei Y. Y., Sun Y. P., Wang B. T. (2020). Woodland strawberry WRKY71 acts as a promoter of flowering via a transcriptional regulatory cascade. *Horticulture Research*.

[42] Chen J. Q., Wang H. P., Li Y., Pan J., Hu Y., Yu D. (2018). ArabidopsisVQ10 interacts with WRKY8 to modulate basal defense againstBotrytis cinerea. *Journal of Integrative Plant Biology*.

[43] Ye Y. J., Xiao Y. Y., Han Y. C. (2016). Banana fruit VQ motif-containing protein5 represses cold-responsive transcription factor MaWRKY26 involved in the regulation of JA biosynthetic genes. *Scientific Reports*.

[44] Lei R. H., Li X. L., Ma Z. B., Lv Y., Hu Y., Yu D. (2017). Arabidopsis WRKY2 and WRKY34 transcription factors interact with VQ20 protein to modulate pollen development and function. *Plant Journal*.

[45] Lei R., Ma Z., Yu D. (2018). WRKY2/34-VQ20 modules in Arabidopsis thaliana negatively regulate expression of a trio of related MYB transcription factors during pollen development. *Frontiers in Plant Science*.

[46] Shirasawa K., Esumi T., Hirakawa H. (2019). Phased genome sequence of an interspecific hybrid flowering cherry, ‘Somei-Yoshino’ (Cerasus × yedoensis). *DNA Research*.

[47] Baek S., Choi K., Kim G. B. (2018). Draft genome sequence of wild Prunus yedoensis reveals massive inter-specific hybridization between sympatric flowering cherries. *Genome Biology*.

[48] Shirasawa K., Isuzugawa K., Ikenaga M. (2017). The genome sequence of sweet cherry (Prunus avium) for use in genomics-assisted breeding. *DNA Research*.

[49] The International Peach Genome Initiative, Verde I., Abbott A. G. (2013). The high-quality draft genome of peach ( _Prunus persica_ ) identifies unique patterns of genetic diversity, domestication and genome evolution. *Nature Genetics*.

[50] Zhebentyayeva T., Shankar V., Scorza R. (2019). Genetic characterization of worldwide _Prunus domestica_ (plum) germplasm using sequence-based genotyping. *Horticulture Research*.

[51] Alioto T., Alexiou K. G., Bardil A. (2020). Transposons played a major role in the diversification between the closely related almond and peach genomes: results from the almond genome sequence. *Plant Journal*.

[52] Ye C. J., Stilgenbauer L., Moy A., Liu G., Heng H. H. (2019). What is karyotype coding and why is genomic topology important for cancer and evolution?. *Frontiers in Genetics*.

[53] Vilanova S., Sargent D. J., Arús P., Monfort A. (2008). Synteny conservation between two distantly-related Rosaceae genomes: Prunus (the stone fruits) and Fragaria (the strawberry). *BMC Plant Biology*.

[54] Jung S., Jiwan D., Cho I. (2009). Synteny of Prunus and other model plant species. *BMC Genomics*.

[55] Dirlewanger E., Graziano E., Joobeur T. (2004). Comparative mapping and marker-assisted selection in Rosaceae fruit crops. *Proceedings of the National Academy of Sciences of the United States of America*.

[56] Illa E., Sargent D. J., Girona E. L. (2011). Comparative analysis of rosaceous genomes and the reconstruction of a putative ancestral genome for the family. *BMC Evolutionary Biology*.

[57] LI Y., ZHONG Y., HUANG K., CHENG Z. M. (2016). Genomewide analysis of NBS-encoding genes in kiwi fruit (Actinidia chinensis). *Journal of Genetics*.

[58] Zhong Y., Guo C., Chu J. J., Liu H., Cheng Z. M. (2018). Microevolution of the VQ gene family in six species of Fragaria. *Genome*.

[59] Wei W., Hu Y., Han Y. T., Zhang K., Zhao F. L., Feng J. Y. (2016). The WRKY transcription factors in the diploid woodland strawberry _Fragaria vesca_ : Identification and expression analysis under biotic and abiotic stresses. *Plant Physiology and Biochemistry*.

[60] Kumar S., Stecher G., Tamura K. (2016). MEGA7: molecular evolutionary genetics analysis version 7.0 for bigger datasets. *Molecular Biology and Evolution*.

[61] Larkin M. A., Blackshields G., Brown N. P. (2007). Clustal W and clustal X version 2.0. *Bioinformatics*.

[62] Xie Z., Wang L., Wang L. (2016). Mutation rate analysis via parent-progeny sequencing of the perennial peach. I. A low rate in woody perennials and a higher mutagenicity in hybrids. *Proceedings of the Biological Sciences*.

[63] Sawyer S. (1989). Statistical tests for detecting gene conversion. *Molecular Biology and Evolution*.

[64] Lamesch P., Berardini T. Z., Li D. H. (2012). The Arabidopsis Information Resource (TAIR): improved gene annotation and new tools. *Nucleic Acids Research*.

[65] Garrido-Gala J., Higuera J. J., Muñoz-Blanco J., Amil-Ruiz F., Caballero J. L. (2019). The VQ motif-containing proteins in the diploid and octoploid strawberry. *Scientific Reports*.

[66] Guo J., Chen J., Yang J., Yu Y., Yang Y., Wang W. (2018). Identification, characterization and expression analysis of the VQ motif-containing gene family in tea plant (Camellia sinensis). *BMC Genomics*.

[67] Shannon P., Markiel A., Ozier O. (2003). Cytoscape: a software environment for integrated models of biomolecular interaction networks. *Genome Research*.

[68] Niu L., Pan L., Zeng W. (2018). Dynamic transcriptomes of resistant and susceptible peach lines after infestation by green peach aphids (Myzus persicae Sülzer) reveal defence responses controlled by the Rm3 locus. *BMC Genomics*.

[69] Alkio M., Jonas U., Declercq M., van Nocker S., Knoche M. (2014). Transcriptional dynamics of the developing sweet cherry ( _Prunus avium_ L.) fruit: sequencing, annotation and expression profiling of exocarp-associated genes. *Horticulture Research*.

[70] Panchy N., Lehti-Shiu M., Shiu S. H. (2016). Evolution of gene duplication in plants. *Plant Physiology*.

[71] Cao Y., Meng D., Abdullah M., Jin Q., Lin Y., Cai Y. (2018). Genome wide identification, evolutionary, and expression analysis of VQ genes from two Pyrus species. *Genes (Basel)*.

[72] Zhang C., Wang D. D., Yang C. H. (2017). Genome-wide identification of the potato WRKY transcription factor family. *PLoS One*.

[73] Brandon C. S., Greenwold M. J., Dudycha J. L. (2017). Ancient and recent duplications support functional diversity of daphnia opsins. *Journal of Molecular Evolution*.

[74] Yang S. H., Zhang X. H., Yue J. X., Tian D., Chen J. Q. (2008). Recent duplications dominate NBS-encoding gene expansion in two woody species. *Molecular Genetics and Genomics*.

[75] Chen Q. M., Li Q. H., Qiao X., Yin H., Zhang S. (2020). Genome-wide identification of lysin motif containing protein family genes in eight Rosaceae species, and expression analysis in response to pathogenic fungus Botryosphaeria dothidea in Chinese white pear. *BMC Genomics*.

[76] Cao Y. P., Han Y. H., Meng D. D. (2018). Expansion and evolutionary patterns of GDSL-type esterases/lipases in Rosaceae genomes. *Functional & Integrative Genomics*.

[77] Zhong Y., Zhang X. H., Shi Q. L., Cheng Z. M. (2021). Adaptive evolution driving the young duplications in six Rosaceae species. *BMC Genomics*.

[78] Soares P., Ermini L., Thomson N. (2009). Correcting for purifying selection: an improved human mitochondrial molecular clock. *American Journal of Human Genetics*.

[79] Ling L., Qu Y., Zhu J. T., Wang D., Guo C. (2020). Genome-wide identification and expression analysis of theVQgene family inCicer arietinumandMedicago truncatula. *PeerJ*.

[80] Wang Q. S., Wang M. H., Zhang X. Z., Hao B., Kaushik S. K., Pan Y. (2011). WRKY gene family evolution in Arabidopsis thaliana. *Genetica*.

[81] Chen J. N., Nolan T. M., Ye H. X. (2017). Arabidopsis WRKY46, WRKY54, and WRKY70 transcription factors are involved in brassinosteroid-regulated plant growth and drought responses. *Plant Cell*.

[82] Li J., Besseau S., Törönen P. (2013). Defense-related transcription factorsWRKY70 andWRKY54 modulate osmotic stress tolerance by regulating stomatal aperture inArabidopsis. *New Phytologist*.

