# Supplementary material for: Recent Duplications Dominate VQ and WRKY Gene Expansions in Six Prunus Species
Source: Int J Genomics. 2021 Dec 17;2021:4066394. doi: 10.1155/2021/4066394 (PMC8710041; doi:10.1155/2021/4066394)
Supplement: Supplementary 1 — Table S1: the correspondence between gene IDs and VQ names in the six Prunus species. [file 4066394.f1.docx]

Supplementary Table S1. The correspondence between gene IDs and VQ names in the six *Prunus* species.

Supplementary Table S1A. The correspondence between gene IDs and VQ names in *Prunus yedoensis*.

| **Gene ID** | **VQ name** |
| --- | --- |
| YA001301 | *PyVQ1* |
| YA006225 | *PyVQ2* |
| YA007887 | *PyVQ3* |
| YA009931 | *PyVQ4* |
| YA010009 | *PyVQ5* |
| YA011282 | *PyVQ6* |
| YA012841 | *PyVQ7* |
| YA014962 | *PyVQ8* |
| YA016390 | *PyVQ9* |
| YA018213 | *PyVQ10* |
| YA101971 | *PyVQ11* |
| YA102758 | *PyVQ12* |
| YA103894 | *PyVQ13* |
| YA200204 | *PyVQ14* |
| YA200480 | *PyVQ15* |
| YA201517 | *PyVQ16* |
| YA201550 | *PyVQ17* |
| YA301005 | *PyVQ18* |
| YA302323 | *PyVQ19* |
| YA400487 | *PyVQ20* |
| YA401176 | *PyVQ21* |
| YA401545 | *PyVQ22* |
| YA401903 | *PyVQ23* |
| YA403570 | *PyVQ24* |
| YA500840 | *PyVQ25* |
| YA502748 | *PyVQ26* |
| YA602554 | *PyVQ27* |
| YA604439 | *PyVQ28* |
| YA604802 | *PyVQ29* |
| YA700260 | *PyVQ30* |
| YA700293 | *PyVQ31* |
| YA700909 | *PyVQ32* |
| YA701074 | *PyVQ33* |
| YA800332 | *PyVQ34* |
| YA802557 | *PyVQ35* |
| YE009685 | *PyVQ36* |
| YE009811 | *PyVQ37* |
| YE009848 | *PyVQ38* |
| YE009885 | *PyVQ39* |
| YE013556 | *PyVQ40* |
| YE102454 | *PyVQ41* |
| YE103205 | *PyVQ42* |
| YE104262 | *PyVQ43* |
| YE104307 | *PyVQ44* |
| YE200169 | *PyVQ45* |
| YE200655 | *PyVQ46* |
| YE200681 | *PyVQ47* |
| YE301023 | *PyVQ48* |
| YE302252 | *PyVQ49* |
| YE302339 | *PyVQ50* |
| YE401212 | *PyVQ51* |
| YE401536 | *PyVQ52* |
| YE403612 | *PyVQ53* |
| YE700266 | *PyVQ54* |
| YE800375 | *PyVQ55* |

Supplementary Table S1B. The correspondence between gene IDs and VQ names in *Prunus domestica*.

| **Gene ID** | **VQ name** |
| --- | --- |
| g0177401 | *PgVQ1* |
| g0209801 | *PgVQ2* |
| g0364901 | *PgVQ3* |
| g0512301 | *PgVQ4* |
| g0718601 | *PgVQ5* |
| g10057001 | *PgVQ6* |
| g10142001 | *PgVQ7* |
| g10178301 | *PgVQ8* |
| g10222601 | *PgVQ9* |
| g10252201 | *PgVQ10* |
| g10842201 | *PgVQ11* |
| g10842501 | *PgVQ12* |
| g10885701 | *PgVQ13* |
| g11030301 | *PgVQ14* |
| g11150501 | *PgVQ15* |
| g11173101 | *PgVQ16* |
| g11720701 | *PgVQ17* |
| g11762801 | *PgVQ18* |
| g1182501 | *PgVQ19* |
| g11991301 | *PgVQ20* |
| g12050901 | *PgVQ21* |
| g12074201 | *PgVQ22* |
| g12074401 | *PgVQ23* |
| g12190701 | *PgVQ24* |
| g12190801 | *PgVQ25* |
| g12273801 | *PgVQ26* |
| g12568701 | *PgVQ27* |
| g12578401 | *PgVQ28* |
| g12874701 | *PgVQ29* |
| g12907401 | *PgVQ30* |
| g13057601 | *PgVQ31* |
| g1582801 | *PgVQ32* |
| g1768801 | *PgVQ33* |
| g2122101 | *PgVQ34* |
| g2133701 | *PgVQ35* |
| g2383101 | *PgVQ36* |
| g2600001 | *PgVQ37* |
| g2917901 | *PgVQ38* |
| g3067601 | *PgVQ39* |
| g3142801 | *PgVQ40* |
| g3217201 | *PgVQ41* |
| g3490801 | *PgVQ42* |
| g3749201 | *PgVQ43* |
| g3767701 | *PgVQ44* |
| g3767801 | *PgVQ45* |
| g3957501 | *PgVQ46* |
| g4014701 | *PgVQ47* |
| g4212201 | *PgVQ48* |
| g4212301 | *PgVQ49* |
| g4672801 | *PgVQ50* |
| g4838501 | *PgVQ51* |
| g5456601 | *PgVQ52* |
| g5487301 | *PgVQ53* |
| g5653001 | *PgVQ54* |
| g6256601 | *PgVQ55* |
| g6348701 | *PgVQ56* |
| g6419501 | *PgVQ57* |
| g6519301 | *PgVQ58* |
| g6577101 | *PgVQ59* |
| g7402601 | *PgVQ60* |
| g7402701 | *PgVQ61* |
| g7574301 | *PgVQ62* |
| g7890801 | *PgVQ63* |
| g8193801 | *PgVQ64* |
| g8335401 | *PgVQ65* |
| g8473201 | *PgVQ66* |
| g8837401 | *PgVQ67* |
| g9730001 | *PgVQ68* |
| g9934901 | *PgVQ69* |
| g9989101 | *PgVQ70* |

Supplementary Table S1C. The correspondence between gene IDs and VQ names in *Prunus avium*.

| **Gene ID** | **VQ name** |
| --- | --- |
| V0000401m | *PvVQ1* |
| V00095100m | *PvVQ2* |
| V0011021m | *PvVQ3* |
| V0025716m | *PvVQ4* |
| V0038331m | *PvVQ5* |
| V0038411m | *PvVQ6* |
| V0039681m | *PvVQ7* |
| V0042824m | *PvVQ8* |
| V0054655m | *PvVQ9* |
| V0062908m | *PvVQ10* |
| V0064913m | *PvVQ11* |
| V0069123m | *PvVQ12* |
| V0075812m | *PvVQ13* |
| V0089009m | *PvVQ14* |
| V0105118m | *PvVQ15* |
| V0110904m | *PvVQ16* |
| V0123613m | *PvVQ17* |
| V0130575m | *PvVQ18* |
| V0136914m | *PvVQ19* |
| V0142846m | *PvVQ20* |
| V0143103m | *PvVQ21* |
| V0154304m | *PvVQ22* |
| V0197407m | *PvVQ23* |
| V0282873m | *PvVQ24* |
| V0370504m | *PvVQ25* |

Supplementary Table S1D. The correspondence between gene IDs and VQ names in *Prunus dulcis*.

| **Gene ID** | **VQ name** |
| --- | --- |
| A000611P1 | *PaVQ1* |
| A001013P1 | *PaVQ2* |
| A003576P1 | *PaVQ3* |
| A006605P1 | *PaVQ4* |
| A006784P1 | *PaVQ5* |
| A007213P1 | *PaVQ6* |
| A009316P1 | *PaVQ7* |
| A009982P1 | *PaVQ8* |
| A010181P1 | *PaVQ9* |
| A010805P1 | *PaVQ10* |
| A011885P1 | *PaVQ11* |
| A013016P1 | *PaVQ12* |
| A013896P1 | *PaVQ13* |
| A019317P1 | *PaVQ14* |
| A019526P1 | *PaVQ15* |
| A020464P1 | *PaVQ16* |
| A021731P1 | *PaVQ17* |
| A023671P1 | *PaVQ18* |
| A024093P1 | *PaVQ19* |
| A027301P1 | *PaVQ20* |
| A028279P1 | *PaVQ21* |
| A032069P1 | *PaVQ22* |
| A032358P1 | *PaVQ23* |

Supplementary Table S1E. The correspondence between gene IDs and VQ names in *Prunus persica*.

| **Gene ID** | **VQ name** |
| --- | --- |
| p1G312500 | *PpVQ1* |
| p1G396600 | *PpVQ2* |
| p1G514600 | *PpVQ3* |
| p1G522100 | *PpVQ4* |
| p1G550700 | *PpVQ5* |
| p2G000100 | *PpVQ6* |
| p2G002600 | *PpVQ7* |
| p2G041200 | *PpVQ8* |
| p3G091700 | *PpVQ9* |
| p3G175400 | *PpVQ10* |
| p3G185800 | *PpVQ11* |
| p4G048900 | *PpVQ12* |
| p4G116000 | *PpVQ13* |
| p4G149000 | *PpVQ14* |
| p4G180100 | *PpVQ15* |
| p4G286700 | *PpVQ16* |
| p5G036600 | *PpVQ17* |
| p5G222000 | *PpVQ18* |
| p6G183200 | *PpVQ19* |
| p6G218200 | *PpVQ20* |
| p6G218300 | *PpVQ21* |
| p6G310500 | *PpVQ22* |
| p6G347000 | *PpVQ23* |
| p7G038200 | *PpVQ24* |
| p7G076600 | *PpVQ25* |
| p8G022500 | *PpVQ26* |

Supplementary Table S1F. The correspondence between gene IDs and VQ names in *Prunus yedoensis* var. *nudiflora*.

| **Gene ID** | **VQ name** |
| --- | --- |
| C0020.20 | *PcVQ1* |
| C0053.11 | *PcVQ2* |
| C0091.4 | *PcVQ3* |
| C0103.68 | *PcVQ4* |
| C0128.13 | *PcVQ5* |
| C0375.15 | *PcVQ6* |
| C0758.8 | *PcVQ7* |
| C0761.18 | *PcVQ8* |
| C1458.10 | *PcVQ9* |
| C1868.5 | *PcVQ10* |
| C2100.6 | *PcVQ11* |
| C2146.40 | *PcVQ12* |
| C2375.1 | *PcVQ13* |
| C2477.70 | *PcVQ14* |
| C2543.2 | *PcVQ15* |
| C2762.15 | *PcVQ16* |
| C2880.2 | *PcVQ17* |
| C2930.23 | *PcVQ18* |
| C3011.6 | *PcVQ19* |
| C3478.6 | *PcVQ20* |
| C3478.5 | *PcVQ21* |
| C3657.12 | *PcVQ22* |
| C3660.11 | *PcVQ23* |
