# Supplementary material for: Recent Duplications Dominate VQ and WRKY Gene Expansions in Six Prunus Species
Source: Int J Genomics. 2021 Dec 17;2021:4066394. doi: 10.1155/2021/4066394 (PMC8710041; doi:10.1155/2021/4066394)
Supplement: Supplementary 2 — Table S2: the correspondence between gene IDs and WRKY names in the six Prunus species. [file 4066394.f2.docx]

Supplementary Table S2. The correspondence between gene IDs and WRKY names in the six *Prunus* species.

Supplementary Table S2A. The correspondence between gene IDs and WRKY names in *Prunus yedoensis*.

| **Gene ID** | **WRKY name** |
| --- | --- |
| YA0023280 | *PyWRKY1* |
| YA0024170 | *PyWRKY2* |
| YA0029240 | *PyWRKY3* |
| YA0031880 | *PyWRKY4* |
| YA0077210 | *PyWRKY5* |
| YA0085480 | *PyWRKY6* |
| YA0096680 | *PyWRKY7* |
| YA0102590 | *PyWRKY8* |
| YA0102980 | *PyWRKY9* |
| YA0112620 | *PyWRKY10* |
| YA0128790 | *PyWRKY11* |
| YA0132960 | *PyWRKY12* |
| YA0137810 | *PyWRKY13* |
| YA0144650 | *PyWRKY14* |
| YA0147000 | *PyWRKY15* |
| YA0148130 | *PyWRKY16* |
| YA0151730 | *PyWRKY17* |
| YA0180300 | *PyWRKY18* |
| YA1010300 | *PyWRKY19* |
| YA1015490 | *PyWRKY20* |
| YA1016760 | *PyWRKY21* |
| YA1017060 | *PyWRKY22* |
| YA1027310 | *PyWRKY23* |
| YA1027320 | *PyWRKY24* |
| YA1028580 | *PyWRKY25* |
| YA1033140 | *PyWRKY26* |
| YA1041110 | *PyWRKY27* |
| YA2023300 | *PyWRKY28* |
| YA2027700 | *PyWRKY29* |
| YA2027740 | *PyWRKY30* |
| YA2033300 | *PyWRKY31* |
| YA2036460 | *PyWRKY32* |
| YA2036470 | *PyWRKY33* |
| YA2039870 | *PyWRKY34* |
| YA2040310 | *PyWRKY35* |
| YA3000410 | *PyWRKY36* |
| YA3001010 | *PyWRKY37* |
| YA3010990 | *PyWRKY38* |
| YA3013060 | *PyWRKY39* |
| YA3021850 | *PyWRKY40* |
| YA3025080 | *PyWRKY41* |
| YA3026310 | *PyWRKY42* |
| YA3031350 | *PyWRKY43* |
| YA3032190 | *PyWRKY44* |
| YA3035720 | *PyWRKY45* |
| YA4001620 | *PyWRKY46* |
| YA4007200 | *PyWRKY47* |
| YA4008090 | *PyWRKY48* |
| YA4010340 | *PyWRKY49* |
| YA4022980 | *PyWRKY50* |
| YA4024740 | *PyWRKY51* |
| YA5012990 | *PyWRKY52* |
| YA5016650 | *PyWRKY53* |
| YA5017560 | *PyWRKY54* |
| YA5020720 | *PyWRKY55* |
| YA5024150 | *PyWRKY56* |
| YA6003670 | *PyWRKY57* |
| YA6004770 | *PyWRKY58* |
| YA6021350 | *PyWRKY59* |
| YA6021550 | *PyWRKY60* |
| YA6030990 | *PyWRKY61* |
| YA6034300 | *PyWRKY62* |
| YA6035700 | *PyWRKY63* |
| YA6038030 | *PyWRKY64* |
| YA6038950 | *PyWRKY65* |
| YA6044220 | *PyWRKY66* |
| YA6044810 | *PyWRKY67* |
| YA6046200 | *PyWRKY68* |
| YA6047570 | *PyWRKY69* |
| YA6049260 | *PyWRKY70* |
| YA7027460 | *PyWRKY71* |
| YA8027830 | *PyWRKY72* |
| YA8031000 | *PyWRKY73* |
| YE0005520 | *PyWRKY74* |
| YE0034360 | *PyWRKY75* |
| YE0045370 | *PyWRKY76* |
| YE0088850 | *PyWRKY77* |
| YE0093400 | *PyWRKY78* |
| YE0104220 | *PyWRKY79* |
| YE0108650 | *PyWRKY80* |
| YE0108660 | *PyWRKY81* |
| YE0109360 | *PyWRKY82* |
| YE0115960 | *PyWRKY83* |
| YE0125000 | *PyWRKY84* |
| YE0125750 | *PyWRKY85* |
| YE0152440 | *PyWRKY86* |
| YE0159030 | *PyWRKY87* |
| YE0165840 | *PyWRKY88* |
| YE0171420 | *PyWRKY89* |
| YE0187290 | *PyWRKY90* |
| YE0187790 | *PyWRKY91* |
| YE0187800 | *PyWRKY92* |
| YE0187820 | *PyWRKY93* |
| YE1003230 | *PyWRKY94* |
| YE1014080 | *PyWRKY95* |
| YE1019430 | *PyWRKY96* |
| YE1020580 | *PyWRKY97* |
| YE1020930 | *PyWRKY98* |
| YE1031760 | *PyWRKY99* |
| YE1031770 | *PyWRKY100* |
| YE1033030 | *PyWRKY101* |
| YE1035210 | *PyWRKY102* |
| YE1037570 | *PyWRKY103* |
| YE2021010 | *PyWRKY104* |
| YE2021050 | *PyWRKY105* |
| YE2021730 | *PyWRKY106* |
| YE2029870 | *PyWRKY107* |
| YE2029880 | *PyWRKY108* |
| YE2033220 | *PyWRKY109* |
| YE2033760 | *PyWRKY110* |
| YE3000240 | *PyWRKY111* |
| YE3000820 | *PyWRKY112* |
| YE3011080 | *PyWRKY113* |
| YE3013230 | *PyWRKY114* |
| YE3022410 | *PyWRKY115* |
| YE3025400 | *PyWRKY116* |
| YE3026510 | *PyWRKY117* |
| YE3031430 | *PyWRKY118* |
| YE3032250 | *PyWRKY119* |
| YE3035990 | *PyWRKY120* |
| YE4001190 | *PyWRKY121* |
| YE4004530 | *PyWRKY122* |
| YE4005450 | *PyWRKY123* |
| YE4007690 | *PyWRKY124* |
| YE4020470 | *PyWRKY125* |
| YE4021790 | *PyWRKY126* |
| YE5014930 | *PyWRKY127* |
| YE5018240 | *PyWRKY128* |
| YE5021190 | *PyWRKY129* |
| YE5024520 | *PyWRKY130* |
| YE6003630 | *PyWRKY131* |
| YE6004770 | *PyWRKY132* |
| YE6019950 | *PyWRKY133* |
| YE6020110 | *PyWRKY134* |
| YE6030830 | *PyWRKY135* |
| YE7020620 | *PyWRKY136* |
| YE8003930 | *PyWRKY137* |
| YE8032540 | *PyWRKY138* |
| YE8034850 | *PyWRKY139* |

Supplementary Table S2B. The correspondence between gene IDs and WRKY names in *Prunus domestica*.

| **Gene ID** | **WRKY name** |
| --- | --- |
| g003210 | *PgWRKY1* |
| g027030 | *PgWRKY2* |
| g028410 | *PgWRKY3* |
| g035860 | *PgWRKY4* |
| g038110 | *PgWRKY5* |
| g041610 | *PgWRKY6* |
| g065670 | *PgWRKY7* |
| g073610 | *PgWRKY8* |
| g073620 | *PgWRKY9* |
| g082450 | *PgWRKY10* |
| g095760 | *PgWRKY11* |
| g1008430 | *PgWRKY12* |
| g1011760 | *PgWRKY13* |
| g1012620 | *PgWRKY14* |
| g1012630 | *PgWRKY15* |
| g1012640 | *PgWRKY16* |
| g1017640 | *PgWRKY17* |
| g1028310 | *PgWRKY18* |
| g1028760 | *PgWRKY19* |
| g1033370 | *PgWRKY20* |
| g1036390 | *PgWRKY21* |
| g1042010 | *PgWRKY22* |
| g1046470 | *PgWRKY23* |
| g1046520 | *PgWRKY24* |
| g104730 | *PgWRKY25* |
| g1047310 | *PgWRKY26* |
| g1047370 | *PgWRKY27* |
| g1051800 | *PgWRKY28* |
| g1054670 | *PgWRKY29* |
| g1055490 | *PgWRKY30* |
| g1057260 | *PgWRKY31* |
| g1057730 | *PgWRKY32* |
| g1061300 | *PgWRKY33* |
| g1065050 | *PgWRKY34* |
| g1069620 | *PgWRKY35* |
| g1071260 | *PgWRKY36* |
| g1071910 | *PgWRKY37* |
| g1073810 | *PgWRKY38* |
| g1074740 | *PgWRKY39* |
| g1081720 | *PgWRKY40* |
| g1087510 | *PgWRKY41* |
| g108760 | *PgWRKY42* |
| g1088930 | *PgWRKY43* |
| g1088940 | *PgWRKY44* |
| g109410 | *PgWRKY45* |
| g109960 | *PgWRKY46* |
| g1099770 | *PgWRKY47* |
| g1111120 | *PgWRKY48* |
| g1111170 | *PgWRKY49* |
| g1111880 | *PgWRKY50* |
| g1118950 | *PgWRKY51* |
| g112260 | *PgWRKY52* |
| g1126510 | *PgWRKY53* |
| g1130280 | *PgWRKY54* |
| g1133600 | *PgWRKY55* |
| g1139300 | *PgWRKY56* |
| g1143780 | *PgWRKY57* |
| g1146560 | *PgWRKY58* |
| g1148370 | *PgWRKY59* |
| g1149190 | *PgWRKY60* |
| g1149200 | *PgWRKY61* |
| g1149210 | *PgWRKY62* |
| g1150710 | *PgWRKY63* |
| g1151600 | *PgWRKY64* |
| g115660 | *PgWRKY65* |
| g1174740 | *PgWRKY66* |
| g1175730 | *PgWRKY67* |
| g1178580 | *PgWRKY68* |
| g1186040 | *PgWRKY69* |
| g1187150 | *PgWRKY70* |
| g1187750 | *PgWRKY71* |
| g1191770 | *PgWRKY72* |
| g1192890 | *PgWRKY73* |
| g119510 | *PgWRKY74* |
| g1195850 | *PgWRKY75* |
| g1200580 | *PgWRKY76* |
| g1214100 | *PgWRKY77* |
| g1214690 | *PgWRKY78* |
| g1214700 | *PgWRKY79* |
| g1216230 | *PgWRKY80* |
| g1218550 | *PgWRKY81* |
| g1230880 | *PgWRKY82* |
| g1234670 | *PgWRKY83* |
| g1237350 | *PgWRKY84* |
| g1243860 | *PgWRKY85* |
| g1244620 | *PgWRKY86* |
| g1254270 | *PgWRKY87* |
| g1255400 | *PgWRKY88* |
| g1258040 | *PgWRKY89* |
| g1264480 | *PgWRKY90* |
| g1269340 | *PgWRKY91* |
| g1291270 | *PgWRKY92* |
| g129280 | *PgWRKY93* |
| g1294920 | *PgWRKY94* |
| g142410 | *PgWRKY95* |
| g143130 | *PgWRKY96* |
| g150090 | *PgWRKY97* |
| g150100 | *PgWRKY98* |
| g150560 | *PgWRKY99* |
| g151160 | *PgWRKY100* |
| g156130 | *PgWRKY101* |
| g157580 | *PgWRKY102* |
| g163810 | *PgWRKY103* |
| g170630 | *PgWRKY104* |
| g170670 | *PgWRKY105* |
| g171440 | *PgWRKY106* |
| g182420 | *PgWRKY107* |
| g182430 | *PgWRKY108* |
| g190040 | *PgWRKY109* |
| g214560 | *PgWRKY110* |
| g214580 | *PgWRKY111* |
| g229800 | *PgWRKY112* |
| g239490 | *PgWRKY113* |
| g240300 | *PgWRKY114* |
| g245070 | *PgWRKY115* |
| g249950 | *PgWRKY116* |
| g250920 | *PgWRKY117* |
| g262410 | *PgWRKY118* |
| g262420 | *PgWRKY119* |
| g262950 | *PgWRKY120* |
| g263830 | *PgWRKY121* |
| g271900 | *PgWRKY122* |
| g277540 | *PgWRKY123* |
| g284550 | *PgWRKY124* |
| g288250 | *PgWRKY125* |
| g293440 | *PgWRKY126* |
| g295790 | *PgWRKY127* |
| g309840 | *PgWRKY128* |
| g314110 | *PgWRKY129* |
| g316590 | *PgWRKY130* |
| g317110 | *PgWRKY131* |
| g325400 | *PgWRKY132* |
| g325690 | *PgWRKY133* |
| g328260 | *PgWRKY134* |
| g329120 | *PgWRKY135* |
| g333140 | *PgWRKY136* |
| g335470 | *PgWRKY137* |
| g335480 | *PgWRKY138* |
| g335490 | *PgWRKY139* |
| g340710 | *PgWRKY140* |
| g362590 | *PgWRKY141* |
| g369460 | *PgWRKY142* |
| g372480 | *PgWRKY143* |
| g372570 | *PgWRKY144* |
| g383900 | *PgWRKY145* |
| g386910 | *PgWRKY146* |
| g402000 | *PgWRKY147* |
| g416650 | *PgWRKY148* |
| g448960 | *PgWRKY149* |
| g456650 | *PgWRKY150* |
| g461910 | *PgWRKY151* |
| g463130 | *PgWRKY152* |
| g464540 | *PgWRKY153* |
| g466010 | *PgWRKY154* |
| g470920 | *PgWRKY155* |
| g475360 | *PgWRKY156* |
| g477650 | *PgWRKY157* |
| g483130 | *PgWRKY158* |
| g485490 | *PgWRKY159* |
| g485500 | *PgWRKY160* |
| g485530 | *PgWRKY161* |
| g504760 | *PgWRKY162* |
| g506020 | *PgWRKY163* |
| g510710 | *PgWRKY164* |
| g518600 | *PgWRKY165* |
| g518610 | *PgWRKY166* |
| g532210 | *PgWRKY167* |
| g532280 | *PgWRKY168* |
| g532290 | *PgWRKY169* |
| g540990 | *PgWRKY170* |
| g541000 | *PgWRKY171* |
| g552810 | *PgWRKY172* |
| g562930 | *PgWRKY173* |
| g564450 | *PgWRKY174* |
| g566940 | *PgWRKY175* |
| g566950 | *PgWRKY176* |
| g566960 | *PgWRKY177* |
| g569970 | *PgWRKY178* |
| g570770 | *PgWRKY179* |
| g570780 | *PgWRKY180* |
| g570790 | *PgWRKY181* |
| g571460 | *PgWRKY182* |
| g572010 | *PgWRKY183* |
| g573090 | *PgWRKY184* |
| g578660 | *PgWRKY185* |
| g585470 | *PgWRKY186* |
| g594420 | *PgWRKY187* |
| g606450 | *PgWRKY188* |
| g608850 | *PgWRKY189* |
| g620270 | *PgWRKY190* |
| g624280 | *PgWRKY191* |
| g626970 | *PgWRKY192* |
| g627990 | *PgWRKY193* |
| g642710 | *PgWRKY194* |
| g644230 | *PgWRKY195* |
| g646320 | *PgWRKY196* |
| g648040 | *PgWRKY197* |
| g648050 | *PgWRKY198* |
| g660880 | *PgWRKY199* |
| g662310 | *PgWRKY200* |
| g664450 | *PgWRKY201* |
| g672140 | *PgWRKY202* |
| g675230 | *PgWRKY203* |
| g678700 | *PgWRKY204* |
| g701060 | *PgWRKY205* |
| g708750 | *PgWRKY206* |
| g716640 | *PgWRKY207* |
| g720650 | *PgWRKY208* |
| g722160 | *PgWRKY209* |
| g744810 | *PgWRKY210* |
| g760170 | *PgWRKY211* |
| g768120 | *PgWRKY212* |
| g776430 | *PgWRKY213* |
| g778380 | *PgWRKY214* |
| g779190 | *PgWRKY215* |
| g782450 | *PgWRKY216* |
| g783270 | *PgWRKY217* |
| g785440 | *PgWRKY218* |
| g791660 | *PgWRKY219* |
| g795880 | *PgWRKY220* |
| g814050 | *PgWRKY221* |
| g821620 | *PgWRKY222* |
| g823810 | *PgWRKY223* |
| g825780 | *PgWRKY224* |
| g826930 | *PgWRKY225* |
| g830460 | *PgWRKY226* |
| g835030 | *PgWRKY227* |
| g836370 | *PgWRKY228* |
| g837690 | *PgWRKY229* |
| g839620 | *PgWRKY230* |
| g867490 | *PgWRKY231* |
| g867500 | *PgWRKY232* |
| g867790 | *PgWRKY233* |
| g867800 | *PgWRKY234* |
| g870530 | *PgWRKY235* |
| g871470 | *PgWRKY236* |
| g874160 | *PgWRKY237* |
| g880110 | *PgWRKY238* |
| g880650 | *PgWRKY239* |
| g882340 | *PgWRKY240* |
| g883900 | *PgWRKY241* |
| g892290 | *PgWRKY242* |
| g893790 | *PgWRKY243* |
| g900960 | *PgWRKY244* |
| g901000 | *PgWRKY245* |
| g917050 | *PgWRKY246* |
| g920180 | *PgWRKY247* |
| g927620 | *PgWRKY248* |
| g931920 | *PgWRKY249* |
| g939950 | *PgWRKY250* |
| g940850 | *PgWRKY251* |
| g949980 | *PgWRKY252* |
| g962060 | *PgWRKY253* |
| g968040 | *PgWRKY254* |
| g968850 | *PgWRKY255* |
| g975700 | *PgWRKY256* |
| g976170 | *PgWRKY257* |
| g979340 | *PgWRKY258* |
| g980710 | *PgWRKY259* |
| g986610 | *PgWRKY260* |
| g991190 | *PgWRKY261* |
| g999010 | *PgWRKY262* |

Supplementary Table S2C. The correspondence between gene IDs and WRKY names in *Prunus avium*.

| **Gene ID** | **WRKY name** |
| --- | --- |
| o1119501b | *PvWRKY51* |
| o5313501m | *PvWRKY52* |
| o6061501b | *PvWRKY53* |
| V0002817m | *PvWRKY1* |
| V0013068m | *PvWRKY2* |
| V0015710m | *PvWRKY3* |
| V00220246m | *PvWRKY4* |
| V0022061m | *PvWRKY5* |
| V0025403m | *PvWRKY6* |
| V00254111m | *PvWRKY7* |
| V00254115m | *PvWRKY8* |
| V0025910m | *PvWRKY9* |
| V0029905m | *PvWRKY10* |
| V0032642m | *PvWRKY11* |
| V0037550m | *PvWRKY12* |
| V00396100m | *PvWRKY13* |
| V0042833m | *PvWRKY14* |
| V0048419m | *PvWRKY15* |
| V00491101m | *PvWRKY16* |
| V0049131m | *PvWRKY17* |
| V0050636m | *PvWRKY18* |
| V0055798m | *PvWRKY19* |
| V0062247m | *PvWRKY20* |
| V0062452m | *PvWRKY21* |
| V0074025m | *PvWRKY22* |
| V0074442m | *PvWRKY23* |
| V0085246m | *PvWRKY24* |
| V0088677m | *PvWRKY25* |
| V0089050m | *PvWRKY26* |
| V0091005m | *PvWRKY27* |
| V0098121m | *PvWRKY28* |
| V0098315m | *PvWRKY29* |
| V0107730m | *PvWRKY30* |
| V0108087m | *PvWRKY31* |
| V0110632m | *PvWRKY32* |
| V0111035m | *PvWRKY33* |
| V0128041m | *PvWRKY34* |
| V0133556m | *PvWRKY35* |
| V0133909m | *PvWRKY36* |
| V0134154m | *PvWRKY37* |
| V0139211m | *PvWRKY38* |
| V01405169m | *PvWRKY39* |
| V0158232m | *PvWRKY40* |
| V0158233m | *PvWRKY41* |
| V0176915m | *PvWRKY42* |
| V0231817m | *PvWRKY43* |
| V0231818m | *PvWRKY44* |
| V0231819m | *PvWRKY45* |
| V0244208m | *PvWRKY46* |
| V0356244m | *PvWRKY47* |
| V0491703b | *PvWRKY48* |
| V0491705m | *PvWRKY49* |
| V0703304m | *PvWRKY50* |

Supplementary Table S2D. The correspondence between gene IDs and WRKY names in *Prunus dulcis*.

| **Gene ID** | **WRKY name** |
| --- | --- |
| A001568P1 | *PaWRKY1* |
| A001657P1 | *PaWRKY2* |
| A001748P1 | *PaWRKY3* |
| A001975P1 | *PaWRKY4* |
| A002073P1 | *PaWRKY5* |
| A004090P1 | *PaWRKY6* |
| A005095P1 | *PaWRKY7* |
| A005633P1 | *PaWRKY8* |
| A005971P1 | *PaWRKY9* |
| A006177P1 | *PaWRKY10* |
| A006215P1 | *PaWRKY11* |
| A007596P1 | *PaWRKY12* |
| A007647P1 | *PaWRKY13* |
| A008567P1 | *PaWRKY14* |
| A009578P1 | *PaWRKY15* |
| A011586P1 | *PaWRKY16* |
| A012059P1 | *PaWRKY17* |
| A012168P1 | *PaWRKY18* |
| A012395P1 | *PaWRKY19* |
| A013051P1 | *PaWRKY20* |
| A013566P1 | *PaWRKY21* |
| A014175P1 | *PaWRKY22* |
| A015240P1 | *PaWRKY23* |
| A015786P1 | *PaWRKY24* |
| A017436P1 | *PaWRKY25* |
| A017718P1 | *PaWRKY26* |
| A018032P1 | *PaWRKY27* |
| A019298P1 | *PaWRKY28* |
| A019680P1 | *PaWRKY29* |
| A020675P1 | *PaWRKY30* |
| A020687P1 | *PaWRKY31* |
| A021325P1 | *PaWRKY32* |
| A021799P1 | *PaWRKY33* |
| A022589P1 | *PaWRKY34* |
| A022742P1 | *PaWRKY35* |
| A022804P1 | *PaWRKY36* |
| A023213P1 | *PaWRKY37* |
| A023218P1 | *PaWRKY38* |
| A023323P1 | *PaWRKY39* |
| A024381P1 | *PaWRKY40* |
| A027240P1 | *PaWRKY41* |
| A027479P1 | *PaWRKY42* |
| A027932P1 | *PaWRKY43* |
| A028352P1 | *PaWRKY44* |
| A028388P1 | *PaWRKY45* |
| A028617P1 | *PaWRKY46* |
| A029053P1 | *PaWRKY47* |
| A030043P1 | *PaWRKY48* |
| A030389P1 | *PaWRKY49* |
| A030482P1 | *PaWRKY50* |
| A030641P1 | *PaWRKY51* |
| A030807P1 | *PaWRKY52* |
| A031132P1 | *PaWRKY53* |
| A031374P1 | *PaWRKY54* |
| A031546P1 | *PaWRKY55* |
| A032520P1 | *PaWRKY56* |

Supplementary Table S2E. The correspondence between gene IDs and WRKY names in *Prunus persica*.

| **Gene ID** | **WRKY name** |
| --- | --- |
| p1G071400 | *PpWRKY1* |
| p1G114800 | *PpWRKY2* |
| p1G223200 | *PpWRKY3* |
| p1G269200 | *PpWRKY4* |
| p1G280700 | *PpWRKY5* |
| p1G283500 | *PpWRKY6* |
| p1G393000 | *PpWRKY7* |
| p1G393100 | *PpWRKY8* |
| p1G407500 | *PpWRKY9* |
| p1G431100 | *PpWRKY10* |
| p1G459100 | *PpWRKY11* |
| p1G564300 | *PpWRKY12* |
| p2G133800 | *PpWRKY13* |
| p2G177400 | *PpWRKY14* |
| p2G177800 | *PpWRKY15* |
| p2G185100 | *PpWRKY16* |
| p2G231300 | *PpWRKY17* |
| p2G264900 | *PpWRKY18* |
| p2G265000 | *PpWRKY19* |
| p2G302500 | *PpWRKY20* |
| p2G307400 | *PpWRKY21* |
| p3G002300 | *PpWRKY22* |
| p3G008600 | *PpWRKY23* |
| p3G098100 | *PpWRKY24* |
| p3G113300 | *PpWRKY25* |
| p3G174300 | *PpWRKY26* |
| p3G202000 | *PpWRKY27* |
| p3G214800 | *PpWRKY28* |
| p3G262100 | *PpWRKY29* |
| p3G270800 | *PpWRKY30* |
| p3G308200 | *PpWRKY31* |
| p4G017600 | *PpWRKY32* |
| p4G066400 | *PpWRKY33* |
| p4G075400 | *PpWRKY34* |
| p4G101100 | *PpWRKY35* |
| p4G217900 | *PpWRKY36* |
| p4G232600 | *PpWRKY37* |
| p5G074200 | *PpWRKY38* |
| p5G106700 | *PpWRKY39* |
| p5G117000 | *PpWRKY40* |
| p5G148700 | *PpWRKY41* |
| p5G187800 | *PpWRKY42* |
| p6G036300 | *PpWRKY43* |
| p6G046900 | *PpWRKY44* |
| p6G168200 | *PpWRKY45* |
| p6G169700 | *PpWRKY46* |
| p6G230600 | *PpWRKY47* |
| p6G244300 | *PpWRKY48* |
| p6G257500 | *PpWRKY49* |
| p6G286000 | *PpWRKY50* |
| p6G294900 | *PpWRKY51* |
| p6G295000 | *PpWRKY52* |
| p6G295100 | *PpWRKY53* |
| p6G345100 | *PpWRKY54* |
| p6G361300 | *PpWRKY55* |
| p7G262600 | *PpWRKY56* |
| p8G230700 | *PpWRKY57* |
| p8G265900 | *PpWRKY58* |

Supplementary Table S2F. The correspondence between gene IDs and WRKY names in *Prunus yedoensis* var. *nudiflora*.

| **Gene ID** | **WRKY name** |
| --- | --- |
| C0002.58 | *PcWRKY1* |
| C0003.40 | *PcWRKY2* |
| C0020.58 | *PcWRKY3* |
| C0020.59 | *PcWRKY4* |
| C0035.57 | *PcWRKY5* |
| C0058.35 | *PcWRKY6* |
| C0072.15 | *PcWRKY7* |
| C0105.43 | *PcWRKY8* |
| C0181.12 | *PcWRKY9* |
| C0223.13 | *PcWRKY10* |
| C0235.16 | *PcWRKY11* |
| C0247.11 | *PcWRKY12* |
| C0286.7 | *PcWRKY13* |
| C0361.39 | *PcWRKY14* |
| C0371.28 | *PcWRKY15* |
| C0404.9 | *PcWRKY16* |
| C0539.9 | *PcWRKY17* |
| C0556.19 | *PcWRKY18* |
| C0623.44 | *PcWRKY19* |
| C0685.30 | *PcWRKY20* |
| C0930.6 | *PcWRKY21* |
| C0937.34 | *PcWRKY22* |
| C1029.1 | *PcWRKY23* |
| C1029.2 | *PcWRKY24* |
| C1060.8 | *PcWRKY25* |
| C1105.7 | *PcWRKY26* |
| C1139.3 | *PcWRKY27* |
| C1227.5 | *PcWRKY28* |
| C1257.7 | *PcWRKY29* |
| C1310.36 | *PcWRKY30* |
| C1336.8 | *PcWRKY31* |
| C1348.13 | *PcWRKY32* |
| C1370.6 | *PcWRKY33* |
| C1376.11 | *PcWRKY34* |
| C1376.12 | *PcWRKY35* |
| C1395.3 | *PcWRKY36* |
| C1450.11 | *PcWRKY37* |
| C1543.7 | *PcWRKY38* |
| C1583.14 | *PcWRKY39* |
| C1669.3 | *PcWRKY40* |
| C1734.4 | *PcWRKY41* |
| C1786.49 | *PcWRKY42* |
| C1836.24 | *PcWRKY43* |
| C1875.1 | *PcWRKY44* |
| C1897.31 | *PcWRKY45* |
| C1969.29 | *PcWRKY46* |
| C2012.2 | *PcWRKY47* |
| C2059.5 | *PcWRKY48* |
| C2060.25 | *PcWRKY49* |
| C2094.17 | *PcWRKY50* |
| C2094.18 | *PcWRKY51* |
| C2323.6 | *PcWRKY52* |
| C2329.1 | *PcWRKY53* |
| C2341.40 | *PcWRKY54* |
| C2523.7 | *PcWRKY55* |
| C2549.4 | *PcWRKY56* |
| C2555.16 | *PcWRKY57* |
| C2645.5 | *PcWRKY58* |
| C2682.11 | *PcWRKY59* |
| C2792.2 | *PcWRKY60* |
| C2906.19 | *PcWRKY61* |
| C3098.10 | *PcWRKY62* |
| C3269.22 | *PcWRKY63* |
| C3318.7 | *PcWRKY64* |
| C3385.1 | *PcWRKY65* |
| C3385.2 | *PcWRKY66* |
| C3395.21 | *PcWRKY67* |
| C3462.10 | *PcWRKY68* |
| C3488.46 | *PcWRKY69* |
| C3488.52 | *PcWRKY70* |
| C3521.12 | *PcWRKY71* |
| C3544.34 | *PcWRKY72* |
| C3692.7 | *PcWRKY73* |
| C3695.3 | *PcWRKY74* |
| C3702.14 | *PcWRKY75* |
| C3742.6 | *PcWRKY76* |
| C3817.27 | *PcWRKY77* |
