# Supplementary material for: Recent Duplications Dominate VQ and WRKY Gene Expansions in Six Prunus Species
Source: Int J Genomics. 2021 Dec 17;2021:4066394. doi: 10.1155/2021/4066394 (PMC8710041; doi:10.1155/2021/4066394)
Supplement: Supplementary 3 — Table S3: the sequence exchange events of VQ and WRKY genes in the six Prunus species. [file 4066394.f3.docx]

Supplementary Table S3. The sequence exchange events of VQ and WRKY genes in the six *Prunus* species.

| **VQ genes** | | |
| --- | --- | --- |
| **Clade** | **Paralogs** | **Orthologs** |
| clade1 | 6 | 5 |
| clade2 | 0 | 1 |
| clade3 | 4 | 6 |
| clade4 | 0 | 14 |
| clade5 | 0 | 0 |
| clade6 | 5 | 28 |
| clade7 | 0 | 0 |
| clade8 | 5 | 40 |
| clade9 | 2 | 7 |
| clade10 | 0 | 0 |
| clade11 | 13 | 44 |
| clade12 | 0 | 9 |
| clade13 | 0 | 31 |
| **Total** | **35** | **185** |

| **WRKY genes** | | |
| --- | --- | --- |
| **Clade** | **Paralogs** | **Orthologs** |
| clade1 | 0 | 0 |
| clade2 | 6 | 33 |
| clade3 | 0 | 0 |
| clade4 | 2 | 15 |
| clade5 | 1 | 15 |
| clade6 | 34 | 97 |
| clade7 | 0 | 0 |
| clade8 | 6 | 2 |
| clade9 | 1 | 131 |
| clade10 | 1 | 22 |
| clade11 | 67 | 154 |
| clade12 | 4 | 6 |
| clade13 | 3 | 51 |
| clade14 | 2 | 8 |
| clade15 | 3 | 8 |
| clade16 | 2 | 58 |
| clade17 | 0 | 0 |
| clade18 | 5 | 4 |
| clade19 | 0 | 0 |
| clade20 | 0 | 3 |
| clade21 | 22 | 96 |
| clade22 | 0 | 0 |
| clade23 | 21 | 79 |
| clade24 | 1 | 9 |
| clade25 | 1 | 31 |
| clade26 | 1 | 10 |
| clade27 | 1 | 1 |
| clade28 | 0 | 0 |
| clade29 | 0 | 19 |
| clade30 | 0 | 0 |
| clade31 | 0 | 0 |
| clade32 | 2 | 1 |
| **Total** | **186** | **853** |
