# Supplementary figures and images for: Recent Duplications Dominate VQ and WRKY Gene Expansions in Six Prunus Species
Source: Int J Genomics. 2021 Dec 17;2021:4066394. doi: 10.1155/2021/4066394 (PMC8710041; doi:10.1155/2021/4066394)

**a**

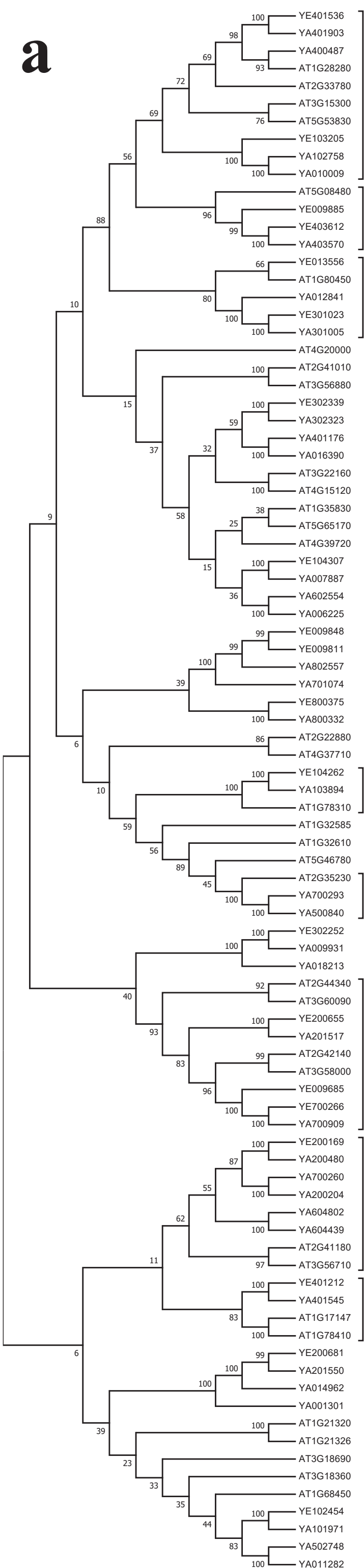

**b**

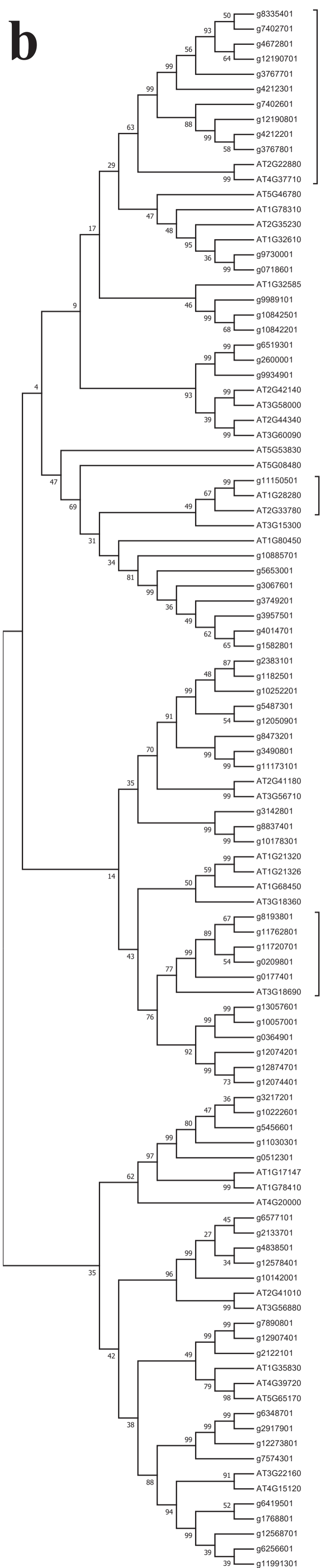

c

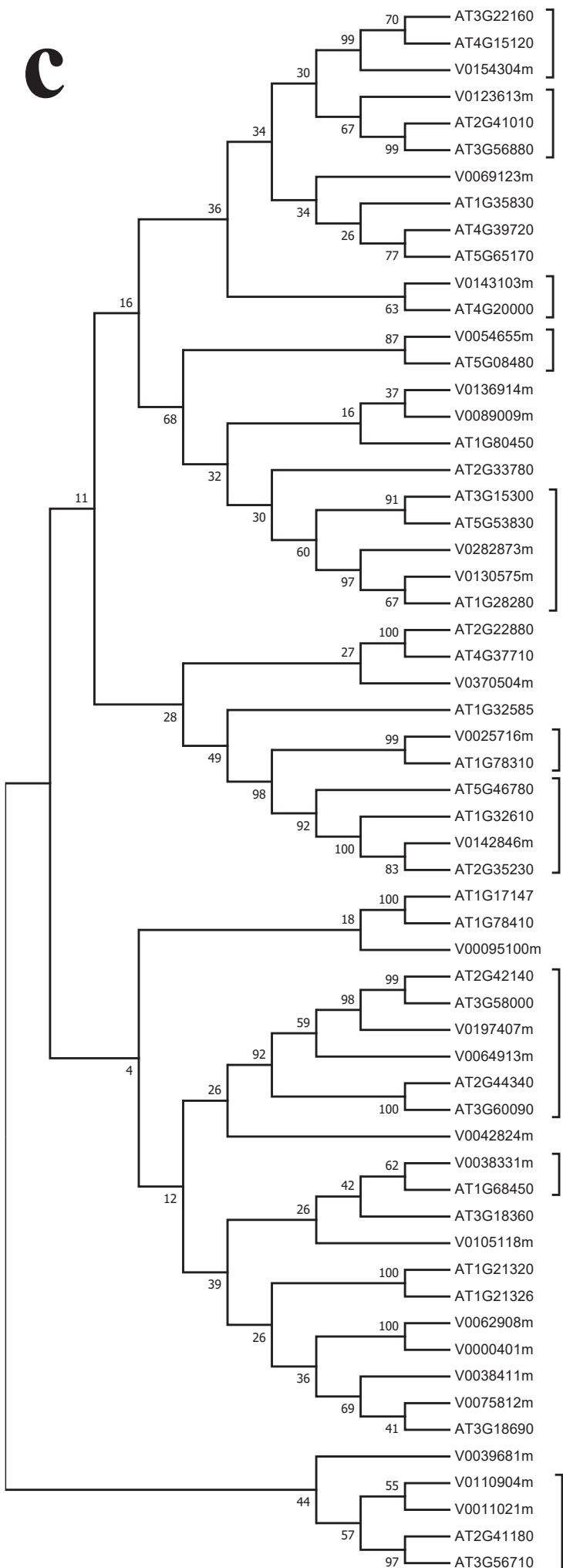

d

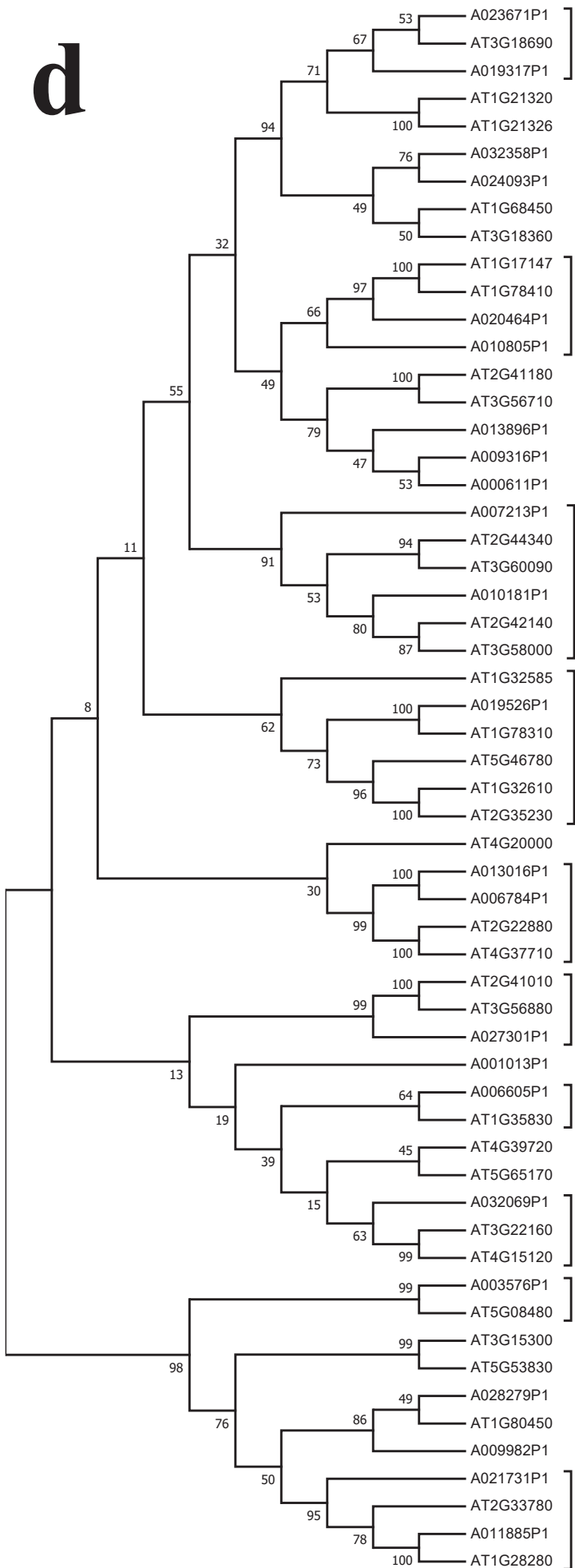

e

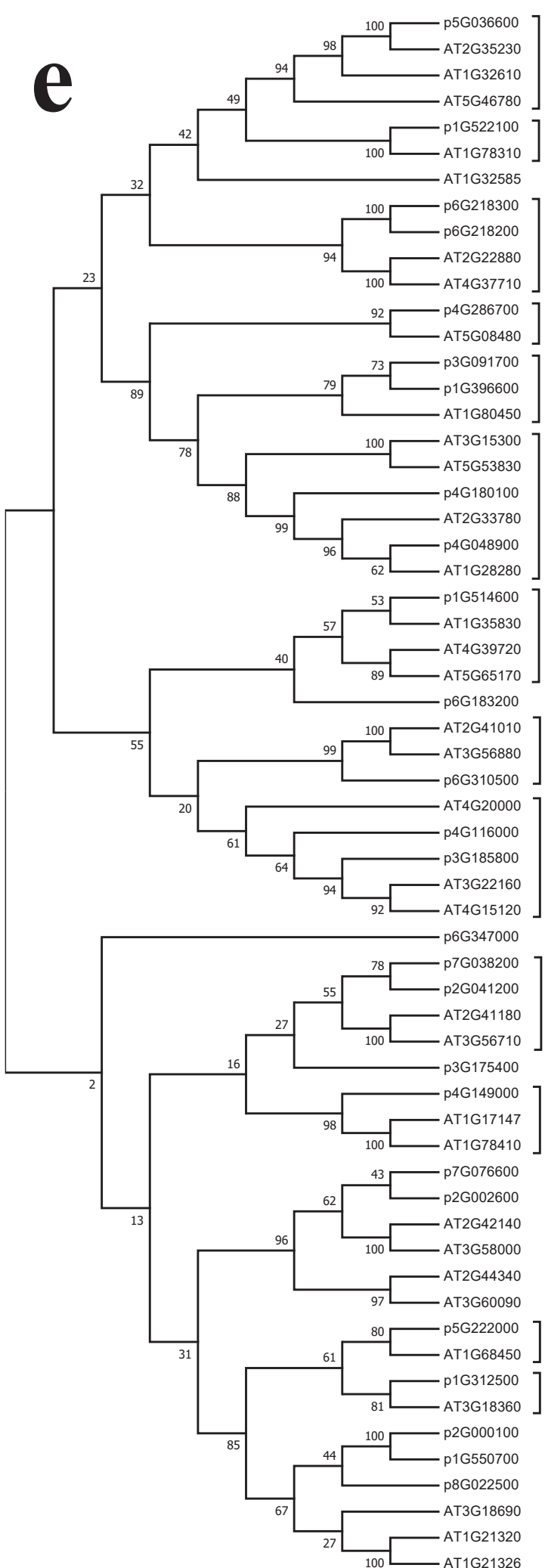

**f**

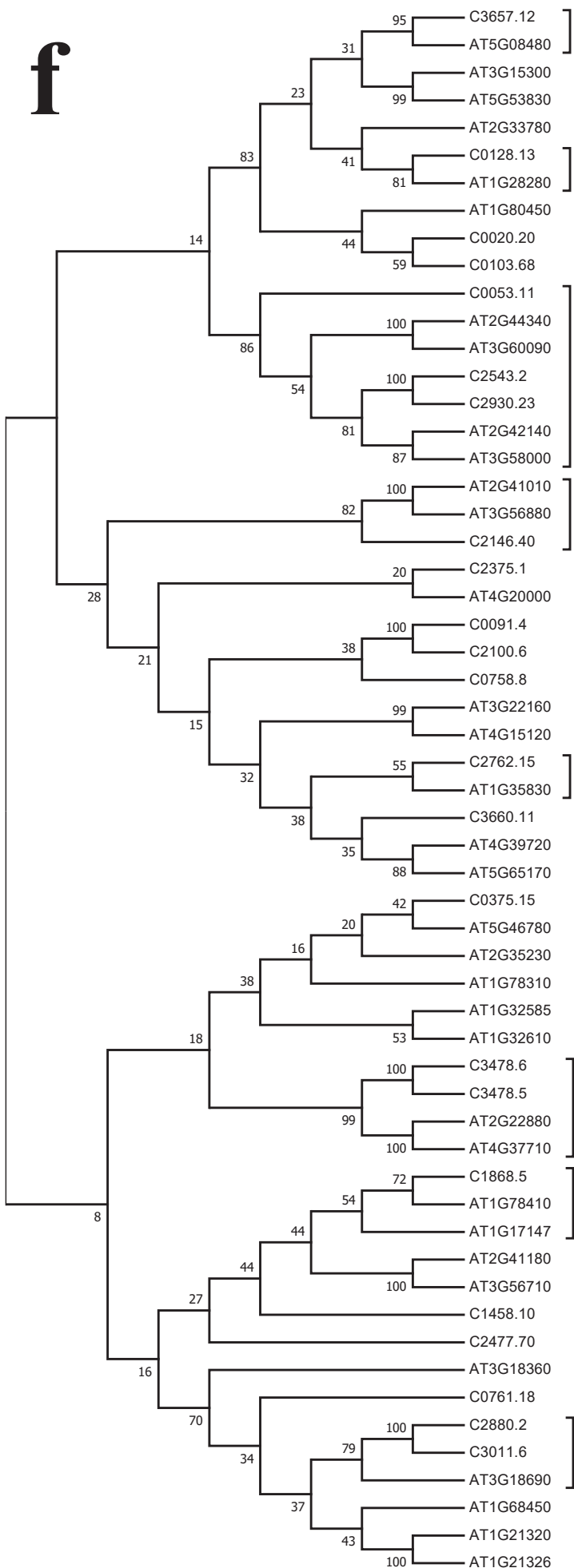

Supplement: Supplementary 4 — Figure S1: phylogenetic trees of VQ genes from Arabidopsis and the six Prunus species. (a) P. yedoensis. (b) P. domestica. (c) P. avium. (d) P. dulcis. (e) P. persica. (f) P. yedoensis var. nudiflora. The square line indicates homologous VQ genes of A. thaliana and Prunus species in the same clade with bootstrap values ≥ 50. [file 4066394.f4.pdf]

**a**

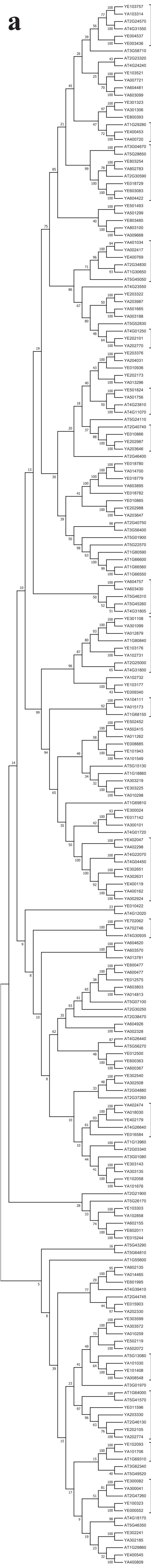

**b**

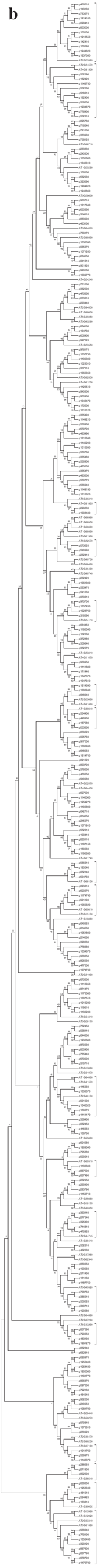

c

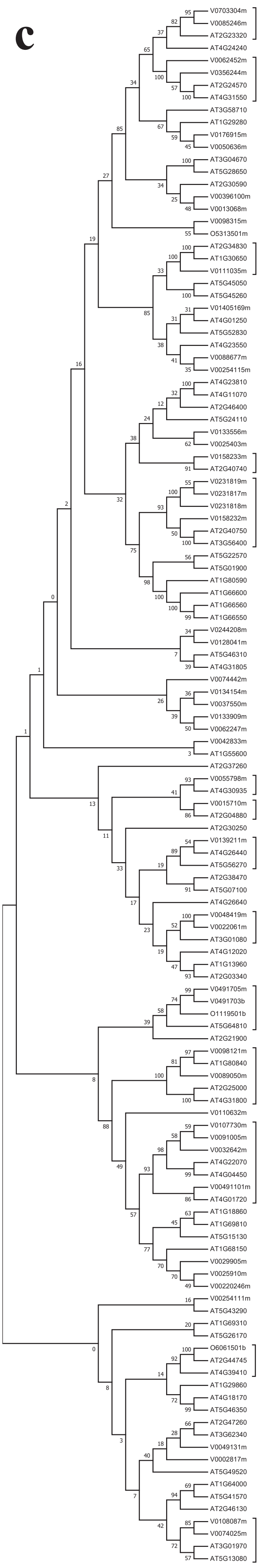

d

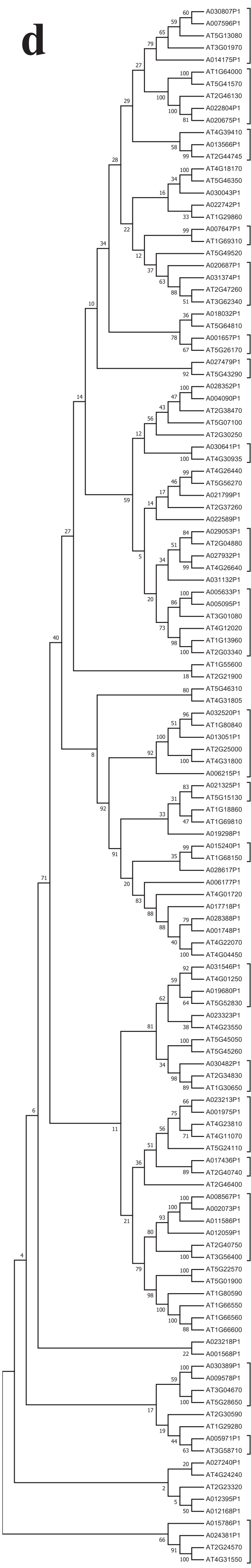

e

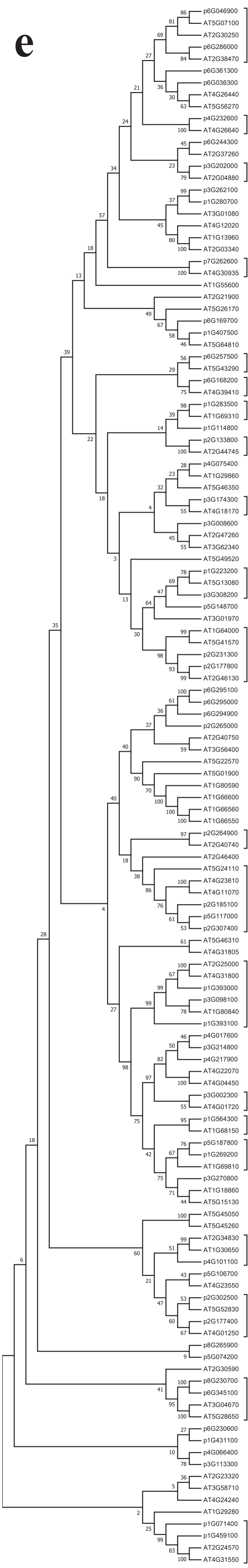

f

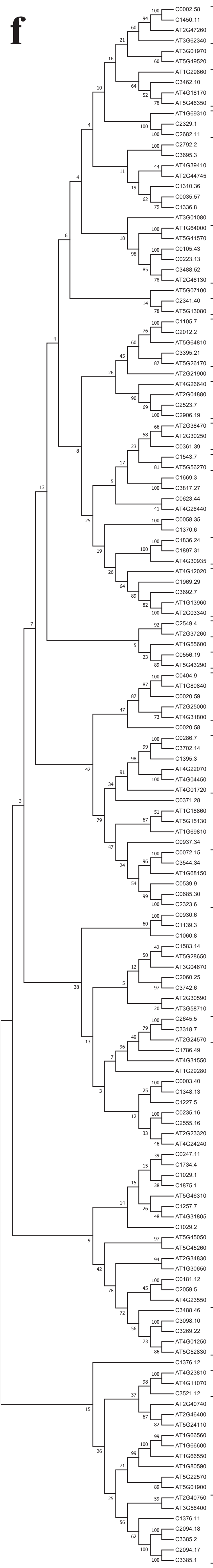

Supplement: Supplementary 5 — Figure S2: phylogenetic trees of WRKY genes from Arabidopsis and the six Prunus species. (a) P. yedoensis. (b) P. domestica. (c) P. avium. (d) P. dulcis. (e) P. persica. (f) P. yedoensis var. nudiflora. The square line indicates homologous WRKY genes of A. thaliana and Prunus species in the same clade with bootstrap values ≥ 50. [file 4066394.f5.pdf]

**a**

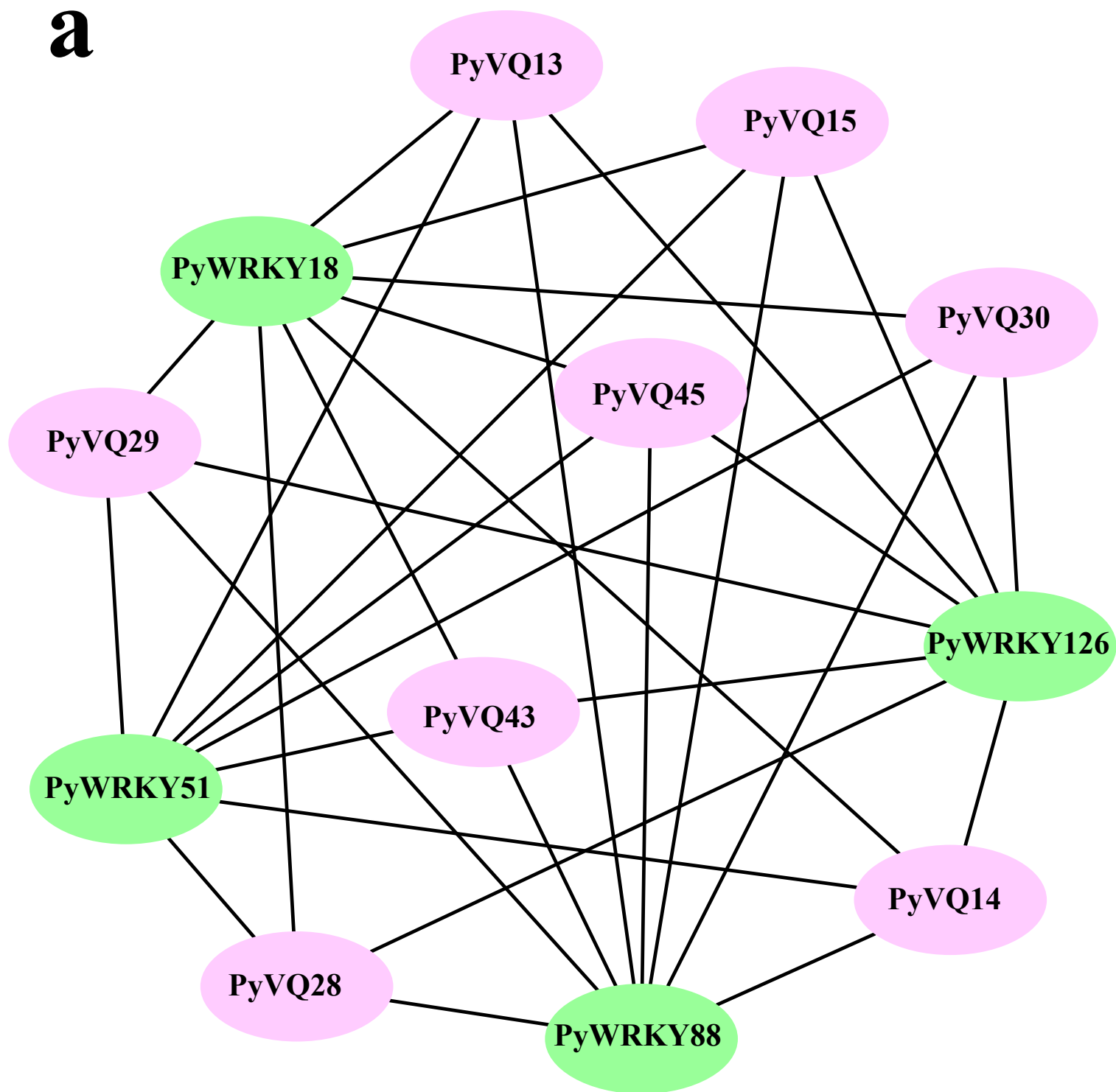

**b**

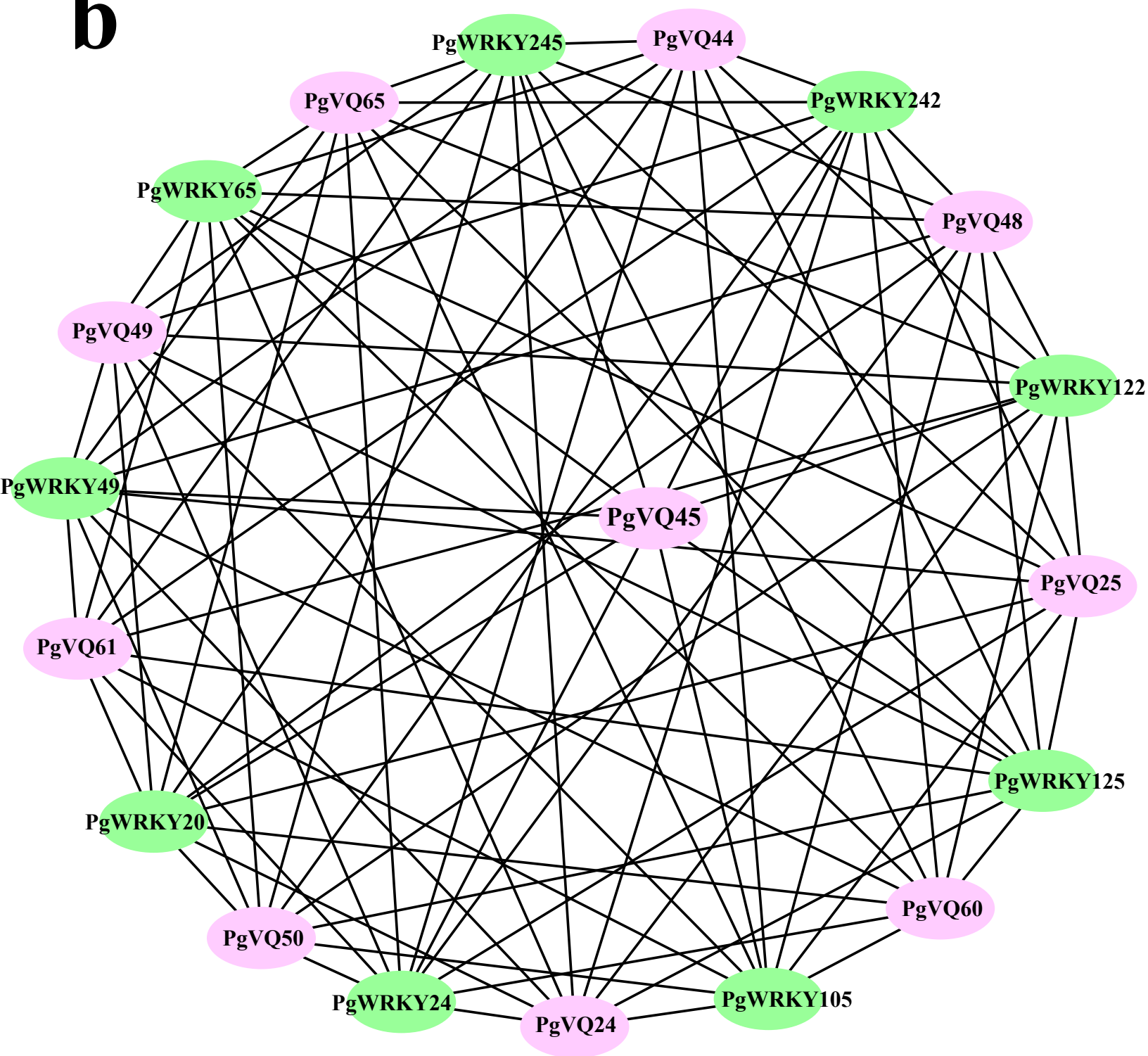

**c**

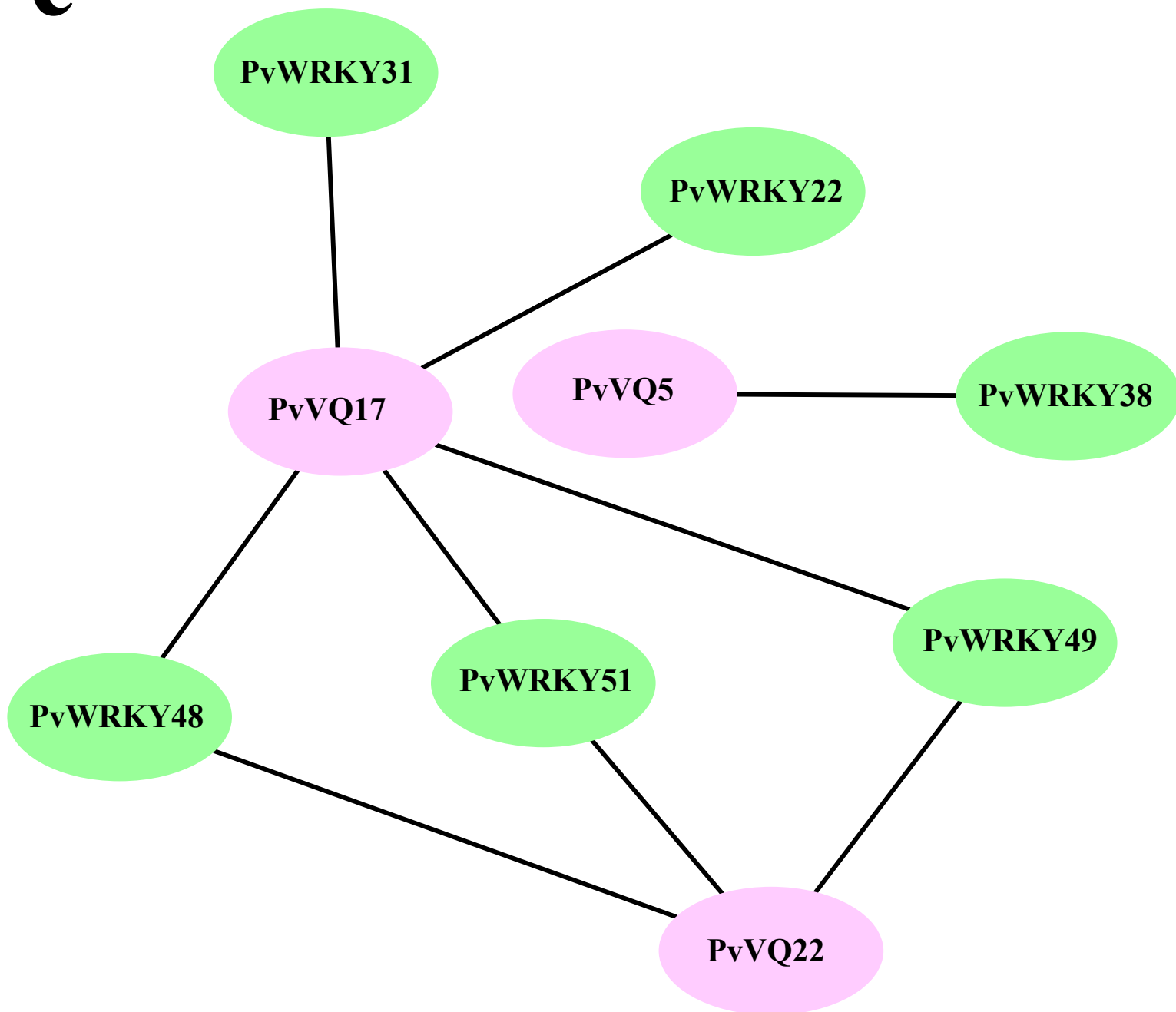

**d**

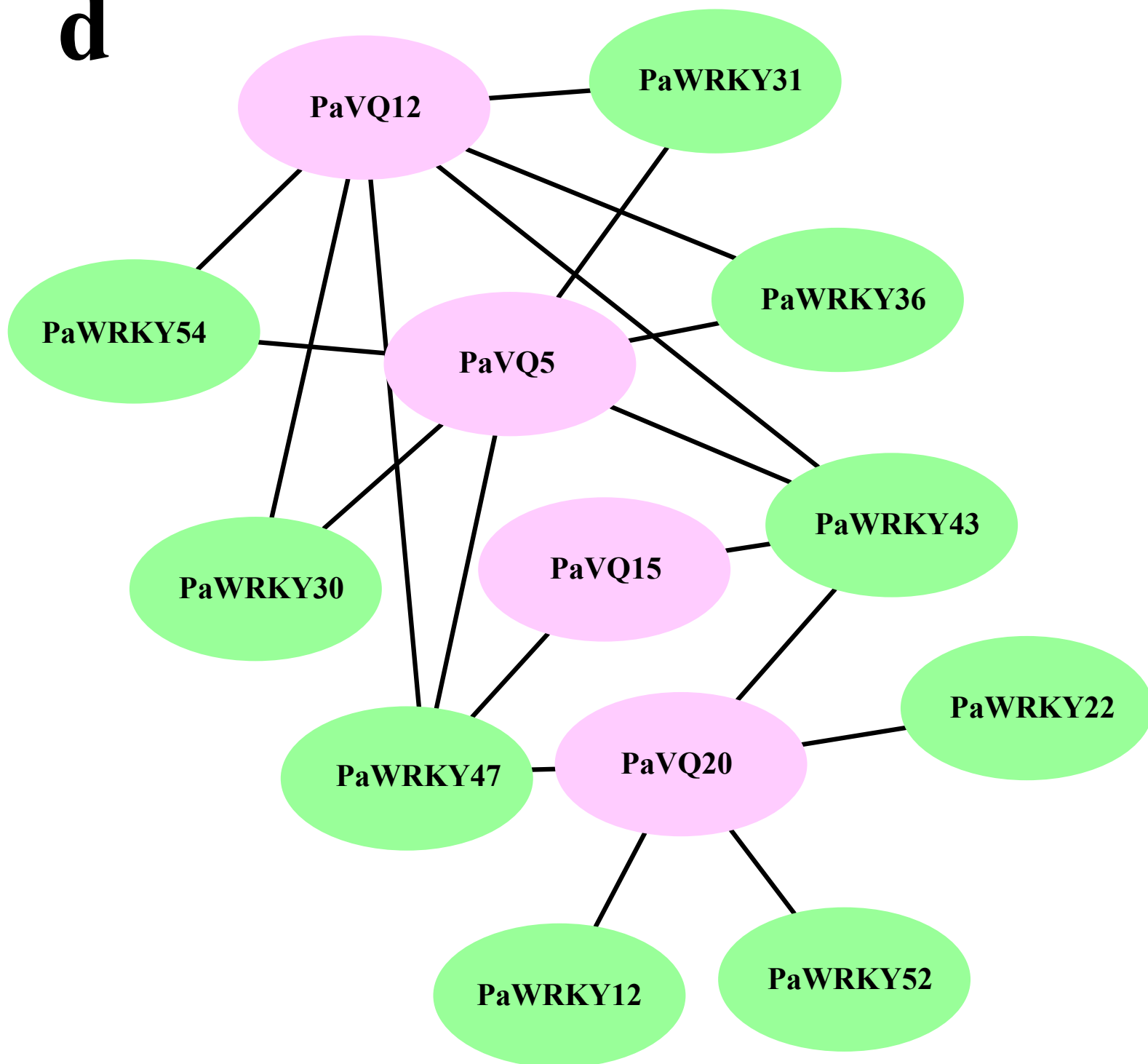

**e**

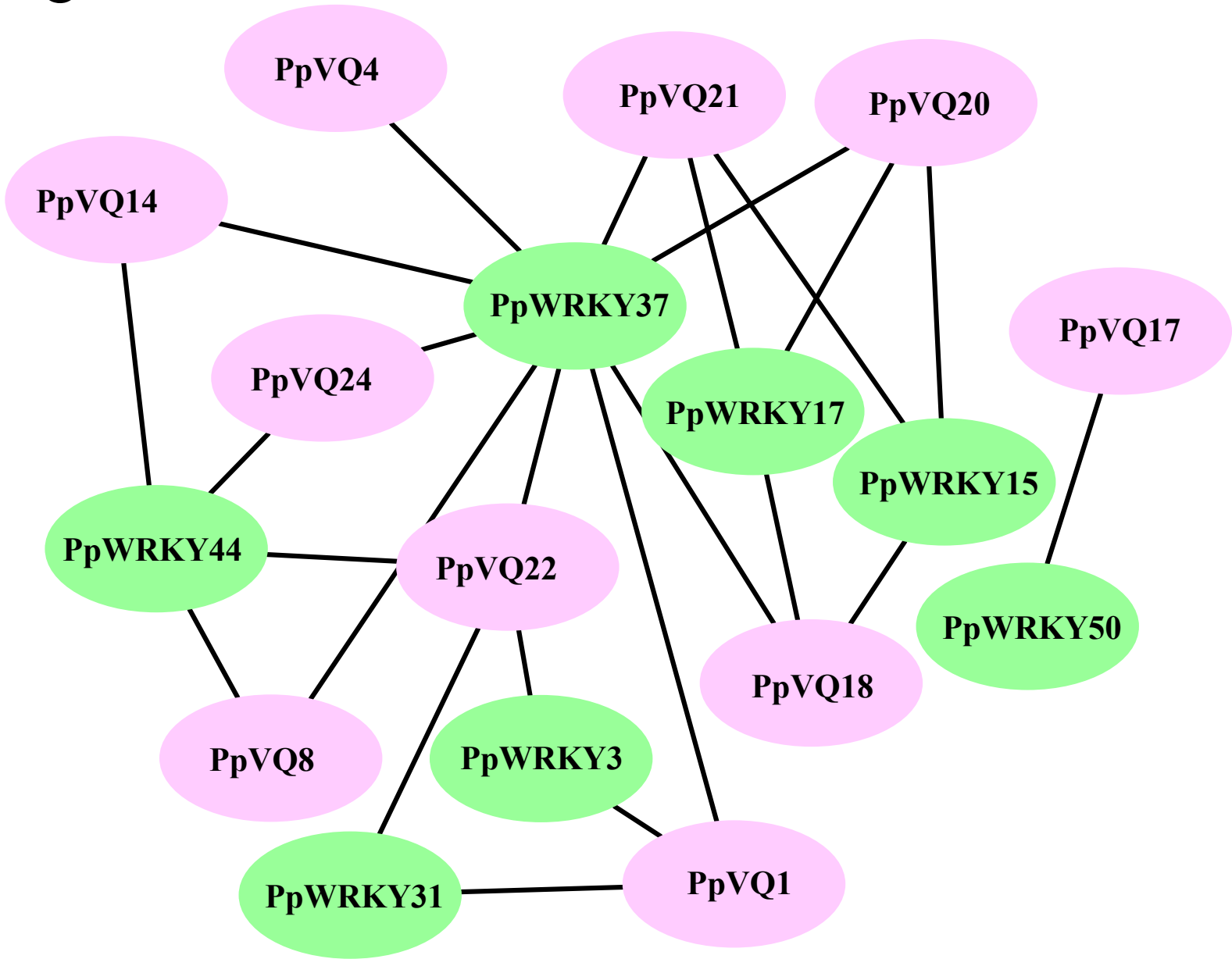

**f**

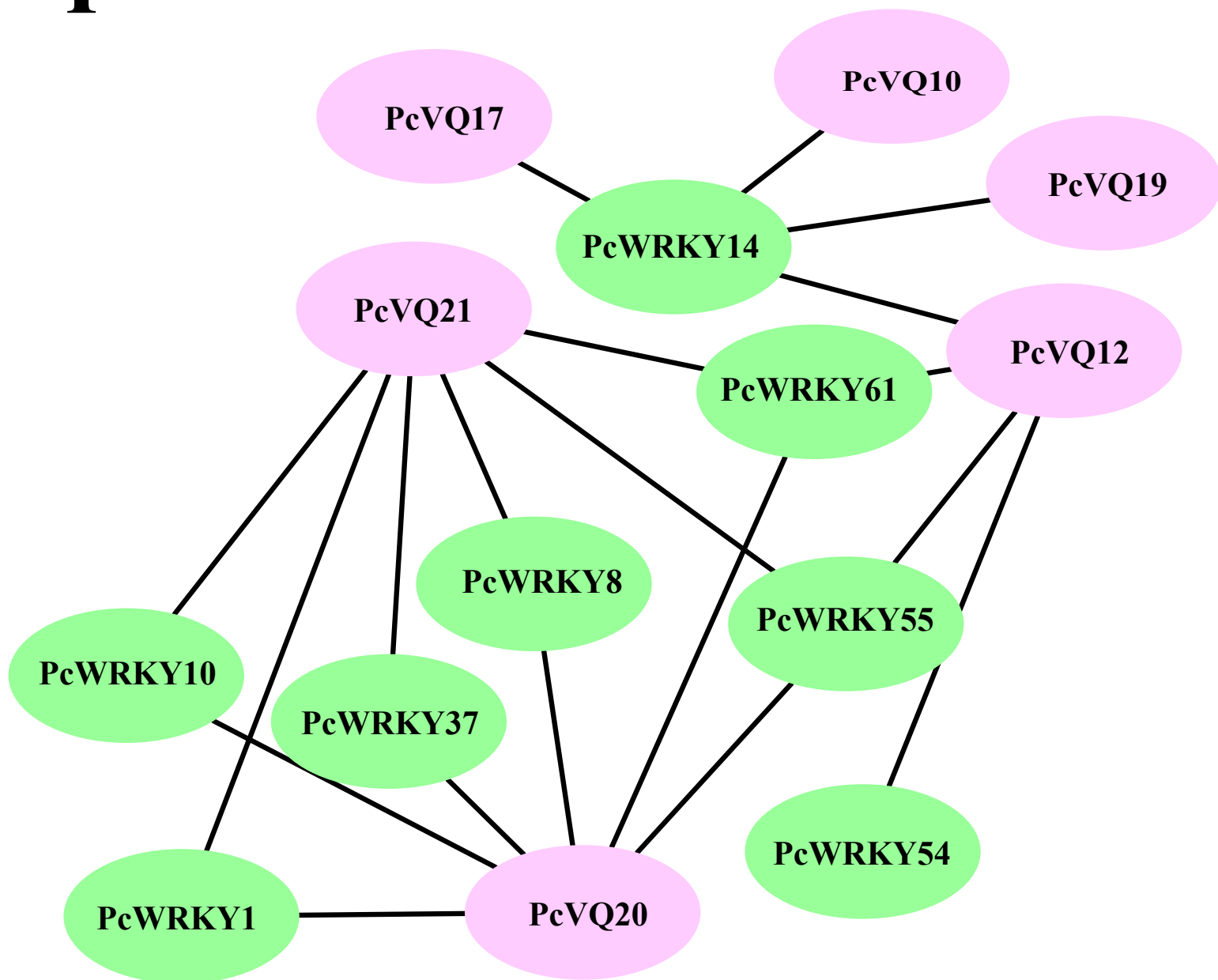

Supplement: Supplementary 6 — Figure S3: predicted interaction networks of VQ and WRKY proteins from the six Prunus species. (a) P. yedoensis. (b) P. domestica. (c) P. avium. (d) P. dulcis. (e) P. persica. (f) P. yedoensis var. nudiflora. [file 4066394.f6.pdf]
